# The pursuit for markers of disease progression in behavioral variant frontotemporal dementia: a scoping review to optimize outcome measures for clinical trials

**DOI:** 10.3389/fnagi.2024.1382593

**Published:** 2024-05-09

**Authors:** Jay L. P. Fieldhouse, Dirk N. van Paassen, Marie-Paule E. van Engelen, Sterre C. M. De Boer, Willem L. Hartog, Simon Braak, Linda J. Schoonmade, Sigfried N. T. M. Schouws, Welmoed A. Krudop, Mardien L. Oudega, Henk J. M. M. Mutsaerts, Charlotte E. Teunissen, Everard G. B. Vijverberg, Yolande A. L. Pijnenburg

**Affiliations:** ^1^Alzheimer Center Amsterdam, Neurology, Vrije Universiteit Amsterdam, Amsterdam UMC Location VUmc, Amsterdam, Netherlands; ^2^Amsterdam Neuroscience, Neurodegeneration, Amsterdam, Netherlands; ^3^Department of Psychiatry, Amsterdam UMC Location VUmc, Amsterdam, Netherlands; ^4^Amsterdam Neuroscience, Mood, Anxiety, Psychosis, Sleep & Stress Program, Amsterdam, Netherlands; ^5^Medical Library, Vrije Universiteit Amsterdam, Amsterdam, Netherlands; ^6^GGZ inGeest Mental Health Care, Amsterdam, Netherlands; ^7^Department of Radiology and Nuclear Medicine, Amsterdam UMC Location VUmc, Amsterdam, Netherlands; ^8^Neurochemistry Laboratory, Department of Laboratory Medicine, Amsterdam UMC Location VUmc, Amsterdam, Netherlands

**Keywords:** literature review, cohort studies, longitudinal, assessment, outcome measures, clinical trials, disease progression

## Abstract

Behavioral variant frontotemporal dementia (bvFTD) is a neurodegenerative disorder characterized by diverse and prominent changes in behavior and personality. One of the greatest challenges in bvFTD is to capture, measure and predict its disease progression, due to clinical, pathological and genetic heterogeneity. Availability of reliable outcome measures is pivotal for future clinical trials and disease monitoring. Detection of change should be objective, clinically meaningful and easily assessed, preferably associated with a biological process. The purpose of this scoping review is to examine the status of longitudinal studies in bvFTD, evaluate current assessment tools and propose potential progression markers. A systematic literature search (in PubMed and Embase.com) was performed. Literature on disease trajectories and longitudinal validity of frequently-used measures was organized in five domains: global functioning, behavior, (social) cognition, neuroimaging and fluid biomarkers. Evaluating current longitudinal data, we propose an adaptive battery, combining a set of sensitive clinical, neuroimaging and fluid markers, adjusted for genetic and sporadic variants, for adequate detection of disease progression in bvFTD.

## 1 Introduction

Behavioral variant frontotemporal dementia (bvFTD), as part of the frontotemporal lobar degeneration (FTLD) spectrum, is a common cause of young-onset dementia (Hogan et al., 2016). Prominent behavioral change is an important feature of bvFTD, including the core behavioral symptoms of disinhibition, apathy, loss of empathy, stereotypy and hyperorality (Rascovsky et al., 2011). BvFTD shows highly variable disease progression (Devenney et al., 2015). Such clinical, pathological and genetic heterogeneity complicates the pursuit for a reliable biomarker of disease progression in bvFTD (Meeter et al., 2017). These different subtypes might require different methods to detect clinical and/or biological progression. However, most instruments used in bvFTD originate from the field of amnestic Alzheimer’s disease and were designed for differential diagnosis with other neurodegenerative diseases, rather than monitor disease progression in bvFTD, let alone its specific subtypes. The fundamental behavioral component in the clinical phenotype of bvFTD calls for a more specific approach. Objective measurement of behavior is complex: behavior is context dependent, observing and reporting of behavior is subjective (to assessor and/or informant) and rarely recognized by the patient itself due to impaired insight (Neary et al., 1998; Mendez and Shapira, 2011). Furthermore, symptomatic overlap with primary psychiatric disorders (PPD), misdiagnosis and diagnostic delay all hamper an adequate characterization of the disease course in bvFTD (Woolley et al., 2011).

A suitable marker for disease progression in bvFTD is highly relevant for both clinical trial design and monitoring disease in clinical practice. To sensitively detect (by)effects of disease modifying therapies, it is crucial to attribute disease severity at baseline (entry status) and measure clinical change during treatment. An ideal outcome measure provides objective, reliable and easy assessment of clinically relevant change that is associated with a biological process. Specificity of possible bvFTD diagnosis is low (Vijverberg et al., 2016; Krudop et al., 2017; de Boer et al., 2023), and certain genetic mutations have been characterized by a typical disease profile, such as mild clinical symptoms and slow disease progression in C9ORF72 mutation carriers (Devenney et al., 2014). Therefore, the identification of disease progression markers in longitudinal cohorts should focus on biomarker confirmed probable or definite bvFTD, preferably, stratifying for genetic mutation status. The purpose of this scoping review is to evaluate the available longitudinal data on clinical [global functioning, behavior, (social) cognition], neuroimaging and fluid biomarkers in bvFTD, in order to identify the most suitable measurements at present, as well as potential needs to be addressed.

## 2 Methods

This scoping review was conducted in accordance with the Preferred Reporting Items for Systematic Reviews and Meta-Analysis (PRISMA) statement [(Page et al., 2021); www.prisma-statement.org]. A comprehensive search was performed in the bibliographic databases PubMed and Embase.com from inception to September 5, 2022, in collaboration with a medical librarian (LS). Search terms included controlled terms (MeSH in PubMed and Emtree in Embase) as well as free text terms. The following terms were used (including all possible synonyms and closely related words) as index terms or free-text words: “behavioral” and “frontotemporal dementia” and “longitudinal studies.” The search was performed without date or language restrictions. Duplicate articles were excluded by a medical information specialist (LS) using Endnote X20.4 (Clarivate^™^), following the Amsterdam Efficient Deduplication (AED) method and the Bramer-method (Bramer et al., 2016; Otten et al., 2019). The full search strategies for all databases can be found in Supplementary Table S1.

Two reviewers (JF and DP) screened all potentially relevant titles and abstracts for eligibility using Rayyan (Ouzzani et al., 2016). Studies resulting from this literature search were included if they met both of the following criteria: (I) population of bvFTD; (II) multiple (follow-up) measurements in time or relevant (cross-sectional) associations with disease progression/severity, to incorporate promising tools currently lacking longitudinal evidence. Studies resulting from this literature search were excluded if they met one or more of the criteria: (I) case-reports; (II) animal studies; (III) reviews; (IIII) focus other than disease progression (e.g., diagnostics). If necessary, the full text article was checked for the eligibility criteria. Two reviewers (JF and DP) evaluated the overall methodological quality of the full text papers taking into account eligibility criteria of (I) high diagnostic accuracy [i.e., probable or definite bvFTD by international diagnostic criteria (Rascovsky et al., 2011)]; (II) sample size; (III) follow-up time; (IIII) use of appropriate outcome measures, when weighing research evidence. Differences in judgement were resolved through a consensus procedure. Literature was organized in five domains: global functioning, behavior, (social) cognition, neuroimaging and fluid biomarkers. These domains were established during the selection procedure to provide structure in the process of identification, evaluation and reporting.

## 3 Results

**Figure 1:**
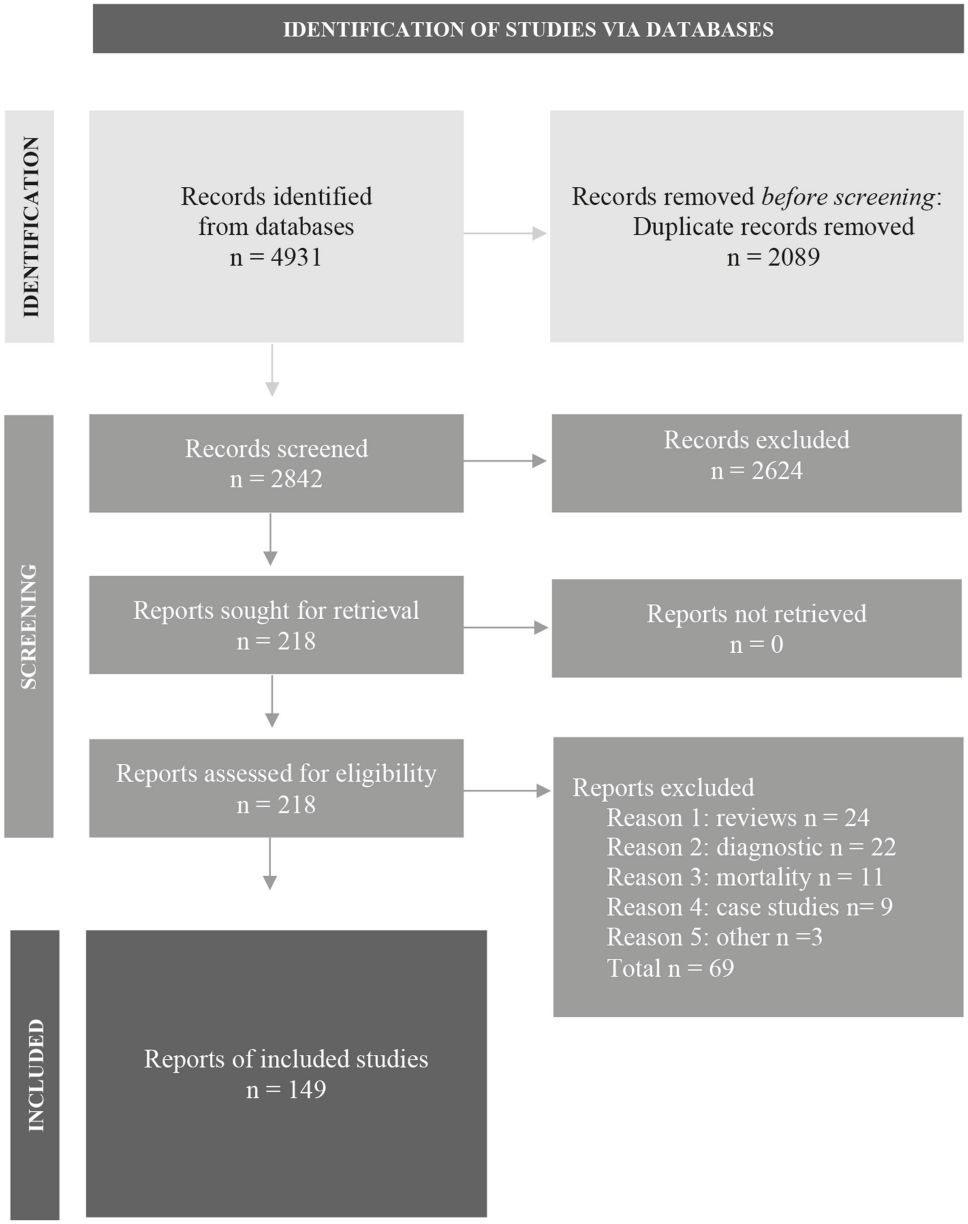
Flowchart of the search and selection procedure of studies.

The literature search generated a total of 4,931 articles: 2,245 in PubMed and 2,686 in Embase. After removing duplicates of articles that were selected from more than one database, 2,842 articles remained. The flow chart of the literature search and selection process is presented in Figure 1 (Page et al., 2021); www.prisma-statement.org. A total of 149 articles were included.

### 3.1 Global functioning

Global rating scales serve as a solid instrument to stage disease severity regardless of underlying neurodegenerative pathology, in a relatively quick and easy manner. Many global dementia scales focus on cognition and do not capture the specific behavioral component in bvFTD. The Clinical Dementia Rating scale (CDR), developed for disease staging of dementia (Morris, 1993), dominated FTD research for many years. By covering mainly Alzheimer’s disease (AD)-related cognitive and functional domains, the CDR tends to underrate disease severity in bvFTD (Mioshi et al., 2010). The adapted version of the CDR, FTLD-modified Clinical Dementia Rating scale (FTLD-CDR); (Knopman et al., 2008), added domains of language and behavior to the original scale, accounting for the most prominent symptoms in bvFTD (Knopman et al., 2011). Findings showed the FTLD-CDR demonstrated annual decline over years in genetic and sporadic FTD population (Knopman et al., 2008; Mioshi et al., 2017; Staffaroni et al., 2019a,b; Anderl-Straub et al., 2021; Lima-Silva et al., 2021). The FTLD-CDR score has been associated with bvFTD specific neuroimaging changes, such as frontotemporal blood flow and atrophy (Borroni et al., 2010; Premi et al., 2016). Therefore, the FLTD-CDR is currently commonly used for disease staging in bvFTD. However, with scores ranging from 0–3, these global rating scales are unable to capture subtle changes, and several other rating scales assess global functioning more extensively. Frequently used scales that measure daily functioning and independence are the Basic Activities of Daily Living (BADL), the Instrumental ADL (IADL), the Disability Assessment for Dementia (DAD), and the Functional Activities questionnaire (FAQ) (Katz et al., 1963; Lawton and Brody, 1969; Pfeffer et al., 1982; Gélinas et al., 1999). Overall, literature demonstrated these measures can detect functional decline in bvFTD over extensive follow-up time (1–7 years) (Knopman et al., 2008; Mioshi and Hodges, 2009; O’Connor et al., 2016; Staffaroni et al., 2019b; Giebel et al., 2021). With regard to behavioral subtypes, a profile of primarily apathy, compared to disinhibition, has been shown to negatively affect daily functioning (DAD) (O’Connor et al., 2017). However, functional autonomy is often preserved up to moderate disease stages, and therefore, FTD-specific scales incorporating the vast behavioral component in bvFTD are more appropriate. As a response, the FTD-Rating Scale (FTD-FRS) was developed to detect both functional dependence and behavioral changes (Mioshi et al., 2010). Longitudinal studies on FTD-FRS captured a multi-domain deterioration over time in sporadic and genetic bvFTD (1–5 years) (Devenney et al., 2015; Schubert et al., 2016; Lima-Silva et al., 2021). The longitudinal outcome measures with most research evidence are listed in Table 1.

**Table 1:** Longitudinal outcome measures with most research evidence in bvFTD.

	Origin	Focus	Pro	Con	Features	Subtypes
**Global functioning**						
FTLD-CDR	FTD (adjusted)	Global rating	▲ ● ♦	Low sensitivity	Rating scale (CR-8)	Genetic and sporadic
FTD-FRS	FTD	Multi-domain	▲ ● ♦	NB. variability	Rating scale (IR-30)	Genetic and sporadic
**Behavior**						
FBI	(FT) Dementia	Multi-domain	▲ ● ■ ♦	NB. variability	Questionnaire (IR-24)	Genetic and sporadic
CBI-R	(FT) Dementia	Multi-domain	▲ ● ■ ♦	NB. variability	Questionnaire (IR-45)	Genetic and sporadic
DAS	Neurodegeneration	Apathy (3D)	● ♦	Lacking follow-up	Questionnaire (PR-24)	Undefined
SRI	FTLD	Stereotypy	● ♦	Limited follow-up	Questionnaire (IR-5)	Undefined
**(Social) cognition**						
ACE-R	(AD) Dementia	Multiple	▲ ●	No social subtest	Screener (PR-26)	Genetic and sporadic
NPA composite scores	Neuro-psychology	Multiple	▲ ●	Time-consuming	Test battery (various)	Genetic and sporadic
NIH-EXAMINER	FTLD	EF and behaviour	● ■ ♦	Limited follow-up	Computer tool (PR-6)	Only genetic
EK-60	Neuro-psychiatry	Emotion recognition	● ■ ♦	Inconsistent FU	Test (PR-60)	Genetic and sporadic
IRI	Neuro-psychology	Empathy	● ■ ♦	Lacking follow-up	Questionnaire (IR-14)	Mostly genetic
RSMS	Neuro-psychology	Socio-emotional	▲● ■ ♦	Limited follow-up	Questionnaire (IR-13)	Mostly genetic
**Neuroimaging**						
MRI ROIs	FTD diagnostics	GM atrophy	▲ ● ♦	Relatively late	Structural (volumetry)	Genetic and sporadic
DTI	Neuro-radiology	WM tract pathology	▲ ● ■ ♦	Time-consuming	Structural (integrity)	Mostly genetic
FDG-PET	FTD diagnostics	Glucose metabolism	▲ ● ■	Low specificity	Functional	Genetic and sporadic
ASL	Neuro-radiology	Cerebral blood flow	● ■	Limited follow-up	Functional	Genetic
**Fluid biomarkers**						
NfL (CSF/serum)	Neuronal damage	Neuro-inflammation	▲ ● ♦	Non specific	CNS mechanism	Genetic and sporadic

Icons of research evidence: ▲ decline over time; ● correlated with disease severity; ■ early abnormalities; ♦ FTD-specific. FTLD-CDR, frontotemporal lobar degeneration modified Clinical Dementia Rating scale; FTD-FRS, Frontotemporal Dementia Rating Scale; FBI, Frontal Behavioral Inventory; CBI-R, Cambridge Behavioral Inventory Revised; DAS, Dimensional Apathy Scale; SRI, Stereotypy Rating Inventory; ACE-R, Addenbrook’s Cognitive Examination Revised; NPA, neuropsychological assessment; NIH-EXAMINER, Neurobehavioral Evaluation & Research; EK-60, Ekman 60-faces test; IRI, Interpersonal Reactivity Index; RSMS, Revised Self-Monitoring Scale; MRI, magnetic resonance imaging; ROIs, regions of interest; DTI, diffusion tensor imaging; FDG-PET, FDG Positron Emission Tomography; ASL, Arterial Spin Labelling; NfL, Neurofilament Light; CSF, cerebrospinal fluid; (bv)FTD, (behavioral variant) frontotemporal dementia; CR, clinician-rated measures (number of items); IR, informant-rated measure (number of items); PR, patient-rated measure (number of items); EF, executive functioning; GM, grey matter; WM, white matter.

### 3.2 Behavior

#### 3.2.1 Important aspects of behavior

Disease progression in bvFTD has been associated with various behavioral changes, from an increase in core features, e.g., decreased socio-emotional abilities and increased multi-dimensional apathy, to specific changes, e.g., increased fat preference and hypersensitivity to loud noises (Midorikawa et al., 2016; Wei et al., 2020; Ahmed et al., 2021; Foster et al., 2022), that correlate with FTD-specific progression measures (FTLD-CDR; FTD-FRS; atrophy rates). Alongside behavior, neuropsychiatric symptoms have been frequently reported, such as depression, anxiety, delusions and hallucinations (Da Silva et al., 2021). For genetic bvFTD, longitudinal cohorts have described mutation-specific behavioral features that seem to be disease phase specified. In early-intermediate phases, MAPT carriers showed increased predominant behavioral symptoms and C9ORF72 carriers showed increased neuropsychiatric symptoms, where after plateauing takes place (Tavares et al., 2020; Benussi et al., 2021b). In late stage on the other hand, C9ORF72 carriers showed decreased reports of depression, whereas GRN carriers showed increased depression and anxiety. Furthermore, behavioral profiles have been associated with age of onset, biological sex and cognitive reserve. Specifically, early onset bvFTD presented with more behavioral symptoms, women showed greater frontotemporal atrophy burden with similar clinical characteristics, and there was a (positive) effect of educational level on rate of change in disinhibition (Linds et al., 2015; Fieldhouse et al., 2021; Illán-Gala et al., 2021a). The concept of behavioral reserve, i.e., behavioral differences in response to a neuropathological burden, was proposed when individuals with less (negative) behavioral symptoms showed a steeper decline in frontotemporal atrophy (Kim et al., 2022). Lastly, it is important to acknowledge the bvFTD phenocopy syndrome (phFTD) as a distinct entity from bvFTD. Apart from clinically mimicking bvFTD while lacking clear etiology, phFTD showed to be non-progressive over an extensive period of time (10+ years) (Devenney et al., 2018).

#### 3.2.2 Behavioral measures

Simply rating the frequency of behavioral criteria and neuropsychiatric symptoms on a 5-point scale was sufficient to detect progression over time in genetic FTD (1–7 years) (Benussi et al., 2021b). However, most frequently used informant-based questionnaires quantify behavioral change more comprehensively. The Neuropsychiatric Inventory (NPI), developed to evaluate psychopathology in AD (Cummings et al., 1994), generally showed increased scores during follow-up in bvFTD (Linds et al., 2015; Da Silva et al., 2021). While parts of AD-oriented neuropsychiatric scales, such as the NPI and the Columbia University Scale for Psychopathology in Alzheimer’s Disease (CUSPAD), predicted cognitive and functional decline in FTD (2.5 years) (Santacruz Escudero et al., 2019), associations with disease severity were inconsistent (Josephs et al., 2011; Kazui et al., 2016; Ranasinghe et al., 2016) and the evidence as bvFTD-specific progression marker was insufficient. The Frontal Behavioral Inventory (FBI) covers a range of FTD-related functional and behavioral symptoms, resulting in a positive (e.g., impulsivity; hyperorality) and a negative symptom score (e.g., lack of empathy; apathy) (Kertesz et al., 1997). Similar to the FBI, the Cambridge Behavioral Inventory-Revised (CBI-R) assesses frequency of FTD-related symptoms (Nagahama et al., 2006; Wear et al., 2008). Literature showed the FBI and the CBI-R to be sensitive to progression in sporadic and genetic bvFTD (C9ORF72) more consistently than the NPI, over varying follow-up time (1–4 years), despite one study stating comparable decline of FBI and NPI (Marczinski et al., 2004; Boutoleau-Bretonniere et al., 2012; Linds et al., 2015; O’Connor et al., 2016; Floeter et al., 2017; Reus et al., 2018). A range of questionnaires that aim to evaluate single behavioral features, currently lacking limited longitudinal validation, may serve as promising progression markers, such as the Dimensional Apathy Scale (DAS) (Radakovic and Abrahams, 2014), assessing three apathy subtypes in neurodegenerative populations, and the Stereotypy Rating Inventory (SRI) quantifying stereotypic and compulsive behaviors in FTLD (Shigenobu et al., 2002). A cross-sectional study on apathy profiles during disease course of bvFTD, showed an increase of DAS scores, while distinguishing emotional apathy in early (<5 years) and executive apathy in later stages (>5 years), associated with distinct neurobiological substrates (Wei et al., 2020). While one study reported no change of stereotypy over time, the SRI predicted progression of frontotemporal atrophy, institutionalization and death (Reus et al., 2018; Gossink et al., 2019) (Table 1).

#### 3.2.3 Course of behavioral symptoms

During disease progression in bvFTD behavioral symptoms may vary, initial behaviors fade whilst new behaviors appear, showing behavioral trajectories are not linear (Diehl-Schmid et al., 2006). The majority of longitudinal studies (including a clinico-pathological study) supported a crescendo-decrescendo trajectory of behavior in bvFTD, in which progressive and diverse behavioral disturbances were followed by dominating apathy (Chow et al., 2012; O’Connor et al., 2016; Borges et al., 2019; Cosseddu et al., 2019). In detail, positive symptoms (such as disinhibition and perseverations) increased until intermediate phases, whereas negative symptoms (such as apathy and loss of empathy) increased throughout disease course. In addition, increased apathy predicted mortality, as stated in a principal component analysis using the Apathy Evaluation Scale (AES), NPI and CBI sub scores (Lansdall et al., 2019). While one study did not report such behavioral inflection point during follow-up (Linds et al., 2015), the relative reduction of positive symptoms may show improvement of behavioral scores over time (Knopman et al., 2008). Similarly, neuropsychiatric symptoms showed to change over time, with symptoms of depression and anxiety in preclinical and prodromal phases, followed by delusions, hallucinations and euphoria in the symptomatic phase (Laganà et al., 2022).

### 3.3 (Social) cognition

#### 3.3.1 Important aspects of cognition

In current international diagnostic criteria, the cognitive profile of bvFTD is characterized by executive deficits, with relative sparing of memory and visuospatial functioning (Rascovsky et al., 2011). However, memory deficits have been increasingly recognized in bvFTD, at initial presentation and over time (Ramanan et al., 2017). A minority of bvFTD (20%) may present with intact cognition at first visit, and thereafter, cognitive decline is variable (Hornberger et al., 2008; Diehl-Schmid et al., 2011; Devenney et al., 2015). For genetic bvFTD, mutation-specific cognitive profiles and trajectories have been identified: characterized decline of confrontational naming, episodic and semantic memory in MAPT carriers, variable deficits (with frequent executive dysfunction) in GRN carriers, and a global and relatively stable profile (e.g., mildly slowed processing speed) in C9ORF72 carriers (Poos et al., 2020; Barker et al., 2021). Pathology-specific profiles point to impaired visual construction in tau-positive FTLD and confrontation naming in tau-negative FTLD, and linguistic deficits in FTLD-TDP (Grossman et al., 2008; Kawakami et al., 2021). Furthermore, multiple studies identified several protective factors of cognitive reserve, i.e., the resilience against neuropathological burden due to lifetime cognitive experiences. Proxies of cognitive reserve included educational level, occupational attainment, late-life social and leisure lifestyle, and specific occupation activities with social skills and cognitive control, which were associated to frontotemporal abnormalities on multiple imaging modalities, including involvement of areas associated to social functioning (prefrontal, anterior temporal and insula) (Dodich et al., 2018; Maiovis et al., 2018; Massimo et al., 2019; Kinney et al., 2021).

#### 3.3.2 Cognitive measures

Cognitive screeners are short, widely used and easily administered instruments to assess global cognition. In bvFTD, most frequently used cognitive screeners are the Mini-Mental State Examination [MMSE; (Folstein et al., 1975)], the Frontal Assessment Battery [FAB; (Dubois et al., 2000)] and, originated as extension of the MMSE, the Addenbrook’s Cognitive Examination Revised [ACE-R; (Mioshi et al., 2006)]. These screeners were not developed for bvFTD, and have proven to be effective in diagnosing or differentiating AD, by emphasizing memory and orientation (MMSE), executive functions (FAB) or briefly covering multiple domains (ACE-R). Literature demonstrated declines of MMSE, FAB and ACE-R total scores in bvFTD (Mioshi and Hodges, 2009; Devenney et al., 2015; Schubert et al., 2016; Reus et al., 2018), but a principal component analysis of these measures (reflecting global cognitive status) showed no association with mortality (Lansdall et al., 2019). For MMSE in specific, rates of decline are known to be lacking or modest, and unrelated to frontotemporal changes on multiple neuroimaging modalities (Borroni et al., 2010; Tan et al., 2013; Premi et al., 2016; Leroy et al., 2021). Due to its comprehensive, yet feasible design, the ACE-R is a more valid cognitive progression screener for bvFTD, with marked rates of decline over follow-up (1–5 years) (Mioshi and Hodges, 2009; Devenney et al., 2015; Schubert et al., 2016). Regarding single tests, the letter fluency detected decline over 18 months in genetic bvFTD (mostly C9ORF72), associated to frontotemporal atrophy and FTLD-CDR progression (Floeter et al., 2016, 2017; Agarwal et al., 2019). However, given cognitive heterogeneity, combining multiple test scores into (executive functioning, language and memory) composites is known to increase sensitivity to change and ability to detect annual decline in bvFTD (Knopman et al., 2008). A combination of ACE-R, executive function and IADL showed to differentiate progressive from non-progressive bvFTD (3 years) (Hornberger et al., 2009). Developed as a clinical trial endpoint, the Executive Abilities: Measures and Instruments for Neurobehavioral Evaluation and Research (NIH-EXAMINER), detected executive and behavioral decline over 18 months in presymptomatic genetic FTD, and was associated with brain volume loss and FTLD-CDR (Staffaroni et al., 2019a) (Table 1). Promising digital tools may increase the sensitivity of cognitive assessment, such as semi-structured speech samples that captured decline of fluency and grammaticality (2 years), associated with frontotemporal atrophy (*N* = 14) (Ash et al., 2019).

#### 3.3.3 Course of cognitive symptoms

Despite cognitive heterogeneity, disease progression in bvFTD has been marked by decline in executive functioning, memory, language and attention (1 to 8 years) (Blair et al., 2007; Wicklund et al., 2007; Smits et al., 2015; Ramanan et al., 2017). The earliest stage was characterized by error insensitivity, slower response time and poor naming, while later stages showed deterioration in a range of executive functions, language and memory, visuo-construction and calculations (Ranasinghe et al., 2016). If impaired at presentation, executive dysfunction was most potent predictor of progression, including grey matter atrophy, institutionalization and mortality (Hornberger et al., 2008; Gossink et al., 2019). Also, language impairment was associated with mortality (Garcin et al., 2009). Studies reported specific patterns of (episodic) memory impairment, with temporal and spatial memory deficits in progressive bvFTD (Irish et al., 2012), and a vulnerability for recent autobiographical memory over time, likely to reflect an encoding deficit rather than retrieval deficit (Irish et al., 2018).

#### 3.3.4 Social cognition

Social cognition deficits are prominent and early features of bvFTD. Social cognition encompasses multiple processes of perceiving, interpreting and regulating social stimuli, including emotion recognition, theory of mind (understanding the cognitive or affective state of others) and social reasoning. Overall, social cognition tests have been well validated for diagnosing bvFTD, but literature on progression is limited. A longitudinal study on emotion recognition, assessed with the Ekman 60-faces test (Aw et al., 2002), reported decline during follow-up (1.5 years), with most rapid decline in bvFTD with marked atrophy (Kumfor et al., 2014). However, other studies did not support this decline, reporting no change or improvement on the Ekman-60-faces over 3 years (Lavenu and Pasquier, 2005; Reus et al., 2018). The addition of different intensities of emotions in the Emotion Recognition Task [ERT; (Kessels et al., 2007)] showed to increase diagnostic sensitivity, even in presymptomatic C9ORF72 carriers (Jiskoot et al., 2021), but longitudinal research is needed. Similarly, first studies on theory of mind (ToM), using different proxies, are inconclusive. One study showed no change of ToM within repeated measures of the Faux Pas test (3 years) (Reus et al., 2018), while performance on Reading the Mind in the Eyes test showed promising associations with disease severity, distinguishing impairment of affective ToM in mild stages from cognitive ToM in severe stages (Torralva et al., 2015). Longitudinal assessment of sarcasm detection, assessed with The Awareness of Social Inference Test [TASIT; (McDonald et al., 2003)], showed a decline in cases with marked atrophy only, indicating it is relatively spared in early stages (Kumfor et al., 2014). Lastly, a cross-sectional study associated distinct social symptoms, as measured by the Social Impairment Rating Scale (SIRS), with three socially relevant (corticolimbic) networks to (Bickart et al., 2014). However, this promising clinician-rated scale requires longitudinal validation. Inconsistent findings in social cognition trajectories highlight current hurdles in the methodology of social cognition assessment, such as possible floor effects due to early impairment and lack of systematic longitudinal multi-level assessment. Novel technologies may improve detection of gradual social cognition decline. Based on the phenomenon of “emotional blunting,” first results on physiological measures (e.g., altered skin conduction or eye gaze) in bvFTD are promising (Joshi et al., 2014; Hutchings et al., 2018; Singleton et al., 2022). Implementation of biometry might capture objective processes related to social functioning (independent of cognitive or cultural factors), highlighting its potential value as (universal) clinical progression marker. Importantly, informant-rated questionnaires on impaired social behavior propose promising markers for progression (Table 1) such as the Revised Self Monitoring Scale (RSMS) and the (modified) Interpersonal Reactivity Index (IRI) (Davis, 1980, 1983; Foster et al., 2022). Socioemotional sensitivity, assessed with the RSMS, showed decline over one year in sporadic and genetic bvFTD, associated to salience network atrophy and caregiver burden (Toller et al., 2020). Yet, correlations between RSMS and social network abnormalities were not supportive, suggesting the true brain-behavior relationship requires further investigation (Toller et al., 2022). Thus far, the IRI, assessing empathetic abilities, was only validated through cross-sectional associations with disease severity (FTLD-CDR) in symptomatic genetic bvFTD, as well as prodromal C9ORF72 carriers (Foster et al., 2022).

### 3.4 Neuroimaging

Since bvFTD is marked by typical frontal and (anterior) temporal atrophy, hypometabolism or hypoperfusion (Rascovsky et al., 2011), the use of neuroimaging offers an essential measure of disease progression. Neuroimaging techniques include a wide range of structural and functional modalities that quantify patterns of grey matter atrophy, white matter integrity, metabolism, perfusion, network connectivity and other processes associated with bvFTD.

#### 3.4.1 Regional atrophy patterns

In general, structural magnetic resonance imaging (MRI) is able to detect frontotemporal grey matter (GM) atrophy patterns during disease progression of bvFTD, by means of quantitative techniques such as voxel-based morphometry (VBM) and deformation-based morphometry (DBM) (Table 1). Whole brain atrophy and ventricular volume increased in both genetic and sporadic bvFTD, in several one-year follow-up studies and one six-month follow-up (Knopman et al., 2009; Gordon et al., 2010; Lam et al., 2014; Floeter et al., 2016; Sheelakumari et al., 2018; Manera et al., 2019; Gordon et al., 2021). Over varying follow-up (from 6 months to 2.5 years), the increase of GM atrophy was associated with various validated clinical measures of disease progression, such as the CDR, CDR-FTD, MMSE, and, in neuropsychological testing, letter fluency scores (Gordon et al., 2010; Floeter et al., 2016; Staffaroni et al., 2019b; Illán-Gala et al., 2021b). Volumetric studies, with mostly extensive follow-up (2.5–5 years), showed fastest progression rates in the temporal lobe (compared with frontal), whereas distinctive regions such as the primary and sensory cortices remain spared (Seeley et al., 2008; Frings et al., 2012; Staffaroni et al., 2019b; Whitwell et al., 2020). However, since many years regional GM atrophy patterns are known to be heterogeneous in bvFTD, of which a cross-sectional study suggested at least four distinct (data-driven) subtypes (Kril et al., 2005; Ranasinghe et al., 2021). Regarding specific regions-of-interest (ROIs), one longitudinal study found a pattern of increased atrophy primarily in the pallidum, middle temporal gyrus, inferior frontal and middle orbitofrontal gyrus, cingulate gyrus and insula over one year (Anderl-Straub et al., 2021). Other ROI-based studies stated the following regions of importance for longitudinal change: anterior cingulate, paracingulate, medial temporal, medial frontal and insular regions (1 year) (Brambati et al., 2007), the medial and lateral frontal lobes, insula, striatum and bilateral temporo-parietal regions (1 year) (Binney et al., 2017), and early and continuing orbitofrontal, anterior cingulate, temporal and subcortical, primarily striatal, regions (1–4 year) (Landin-Romero et al., 2017). Specific regions have been correlated with decline on clinical measures, such as (left) striatum atrophy and the FTLD-CDR and FBI negative subscale (cross-sectional) (Macfarlane et al., 2015), posterior parietal atrophy and loss of recent autobiographical memory over one year (Irish et al., 2018), and olfactory bulb atrophy (specific to more severe disease stages) and olfactory dysfunction (loss of smell) over 1 year (Carnemolla et al., 2022).

#### 3.4.2 White matter integrity patterns

A relatively large amount of studies on diffusion tensor imaging (DTI), visualizing the microstructure of white matter (WM) tracts, concluded sensitive detection of WM changes in an early phase of the disease, over varying follow-up time (0.5 to 2.5 years) (Mahoney et al., 2015; Elahi et al., 2017; Floeter et al., 2018; Kassubek et al., 2018; Staffaroni et al., 2019b). DTI may detect bvFTD pathology before GM atrophy arises, and has been correlated with cognitive decline (cross-sectional ACE-R), contributing to its value as possible early and sensitive progression marker (Chen and Kantarci, 2020) (Table 1). More general, WM tract pathology can be measured by multiple techniques. It’s microtructural integrity can be detected by diffusion-weighted imaging (DWI), of which DTI is a relevant modality as it enables the tracking of WM-fibers (tractography). Macro-structurally, WM pathology can be measured by structural MRI. Progression of WM microstructural disintegrity, as detected by DTI, showed fast rates in early bvFTD (1 year) (Lam et al., 2014). WM volume, as measured with structural MRI, manifested a steeper decline, especially in the temporal lobe, compared to early GM orbitofrontal and insula atrophy (1 year, *N* = 15) (Frings et al., 2014). WM pathology has been correlated with a decline in executive functioning (1 year) (Yu and Lee, 2019), the presence of a MOPB-risk allele (Massimo et al., 2021) and an increase of WM hyperintensities, both independent of and related to cortical atrophy (cross-sectional) (Huynh et al., 2021). In contrast, one cross-sectional study on a clinically relevant outcome measure (Revised Self-Monitoring Scale), found that GM volumes of the right orbitofrontal cortex, not WM tract pathology (DWI), predicted socioemotional impairment (Toller et al., 2022).

#### 3.4.3 Changes in metabolism, perfusion and network connectivity

A prospective study on glucose metabolism (fludeoxyglucose-positron emission tomography; FDG-PET) indicated a specific progression pattern over 1.5 years, from decreased glucose uptake in frontal lobe(s), to parietal and temporal lobe(s), to whole frontal lobe hypometabolism (Diehl-Schmid et al., 2007) (Table 1). A genetic study on arterial spin labelling (ASL) in FTD patients, measuring cerebral blood flow (CBF), showed that a specific pattern of frontal, temporal, parietal and subcortical CBF decrease accompanied the clinical conversion from pre-symptomatic to symptomatic stages in MAPT and GRN mutation carriers over 2 years (Dopper et al., 2016). Multiple promising, yet cross-sectional, studies on single photon emission computer tomography (SPECT) reported a decrease in regional CBF in bilateral frontal cortices and right temporal cortices that correlated with several clinical measures, such as the FTLD-CDR, FTD-FRS, and cognitive reserve scales (Borroni et al., 2010; Maiovis et al., 2017, 2018), as well as specific brainstem hypoperfusion that associated with fast clinical progression in bvFTD (Le Ber et al., 2006). Connectivity changes of the salience network (SN), related to the fundamental behavioral and socioemotional deficits in bvFTD, may be measured with functional MRI (fMRI). Although only reported in a small study with limited longitudinal data (8 weeks), specific SN connectivity patterns (e.g., decreased right fronto-dorsal SN) were associated with increased apathy measured with FBI (Day et al., 2013). While lacking longitudinal data, two small yet promising cross-sectional studies on disruption of sensory/auditory information processing, as measured by magnetoencephalography (MEG) analysis of cortical microcircuits, suggested these changes in frontotemporal networks may be a useful biomarker to detect (early) disease progression (2013, *N* = 12, 2019, *N* = 44) (Hughes and Rowe, 2013; Shaw et al., 2019).

#### 3.4.4 Other pathological processes

While studied in limited follow-up or cross-sectional designs, additional PET and MRI-based techniques, focusing on other pathological processes may hold promise as biomarkers of disease progression. First, a small prospective study (*N* = 10) detected progression of tau-pathology by means of flortaucipir-PET in the frontotemporal region after 1.5 months, and suggested that FTD-specific (tau) tracers could potentially be of superior value (Tsai et al., 2019). Second, a couple of cross-sectional studies detected processes of synaptic loss (11C-UCB-J-PET, *N* = 11) (Malpetti et al., 2021, 2022), and reduced brain stiffness, which is hypothesized to occur prior to gliosis and cellular damage (magnetic resonance elastography, *N* = 5) (Huston et al., 2016). Both processes may be associated with early disease progression in bvFTD.

### 3.5 Fluid biomarkers

Most validated fluid biomarkers are primarily used to differentiate bvFTD from AD, other neurodegenerative disease, or PPD, without being able to accurately diagnose or sensitively monitor bvFTD itself. Current methods do not yet enable *in vivo* quantification of bvFTD pathologies, i.e., aggregation and accumulation of abnormal protein inclusions, primarily tau, TAR DNA-binding protein 43 (TDP-43) or FUS. However, the use of fluid biomarkers may reveal processes that lay closest to pathogenesis and progression of disease, and significant progress has been made. Genetic bvFTD, associated with mutation-related proteinopathies (tau in MAPT, and TDP-43 in GRN and C9ORF72), may serve as a solid base to predict underlying pathology and disease mechanisms. Since this is not yet possible in sporadic bvFTD, similar techniques may ultimately facilitate prediction of underlying pathology in the sporadic variant too. Detection of several fluid biomarkers, through cerebrospinal fluid (CSF) or, less invasive, through serum/plasma, may enable an evaluation of underlying proteinopathies and various downstream effects of neurodegeneration.

#### 3.5.1 Biomarkers indicative of underlying proteinopathies

To date, no fluid biomarkers are known that enable specific detection of bvFTD. A first prospective study on a bvFTD specific proteinopathy related to progranulin (PGRN), which is a protective protein altered in GRN mutation carriers which results in pathological TDP-43 accumulation, showed no significant change in CSF or serum PGRN levels at one-year follow-up (Feneberg et al., 2016). Despite apparent variability, PGRN concentrations did decrease in four out of five FTD patients, calling for further large scale investigation. Next to this, CSF amyloid-beta, which is typically decreased in AD, showed to decrease in both genetic and sporadic bvFTD over five year follow-up, and has been associated with higher mortality (Vieira et al., 2019). Cross-sectional studies on other AD-related proteins showed alternations in bvFTD as well, such as plasma tau and the phosphorylated-tau/total-tau ratio (Foiani et al., 2018; Meeter et al., 2018). However, since these protein profiles are not specific to bvFTD, and did not correlate with important progression measures such as whole brain volume, GM atrophy, neurofilament light chain (NfL), or disease duration, they do not have much potential to measure disease progression (Foiani et al., 2018; Meeter et al., 2018).

#### 3.5.2 Downstream effects of neurodegeneration

Currently, the most promising fluid biomarker, measured in both CSF and serum, is neurofilament light chain (NfL), reflecting axonal damage (Table 1). Longitudinal studies, with 9 to 12 months follow-up, concluded levels of CSF or serum NfL increased over time, in both genetic and sporadic bvFTD (Ljubenkov et al., 2018; Gendron et al., 2022). Additionally, serum NfL was found to predict clinical conversion from a prodromal to a symptomatic phase in a genetic bvFTD cohort at one-year follow-up (Benussi et al., 2021a). Increased CSF NfL, in both genetic and sporadic subtypes, has been associated with various progression measures, including CDR, cognition (executive functioning; neuropsychiatry unit cognitive assessment tool), behavioral symptoms (FBI), frontotemporal GM atrophy, WM tract pathophysiology, GABA-ergic deficit, and survival rates (Scherling et al., 2014; Kassubek et al., 2018; Steinacker et al., 2018; Benussi et al., 2020; Spotorno et al., 2020; Walia et al., 2022). Interestingly, when comparing genetic and sporadic subtypes, a large cross-sectional study concluded that serum NfL concentration is higher in genetic bvFTD (Benussi et al., 2022). Another promising, less validated fluid biomarker is soluble triggering receptor expressed on myeloid cells 2 (sTREM2). Also interpreted as a more general response to neuronal injury, first cross-sectional results showed CSF sTREM2 levels increased during neuro-inflammation in familial bvFTD associated with GRN mutations (*N* = 3) (Woollacott et al., 2018). Contrarily, first cross-sectional results on glial fibrillary acidic protein (GFAP), suggesting to reflect reactive astrogliosis, showed less promising results as suitable progression marker in genetic and sporadic bvFTD, since merely small changes in serum concentration of GFAP were detected (cross-sectional) (Oeckl et al., 2022). The neurotransmitter orexin A, known for regulation of various physiological functions (such as appetite and sleep), has been correlated with obsessive-compulsive (measured by SRI) and extrapyramidal symptoms, that may accompany disease progression (cross-sectional, *N* = 40) (Roveta et al., 2022). Lastly, specific metabolic changes were found in bvFTD (compared to controls), such as altered metabolites in a wide range of pathways (including amino acids, energy and carbohydrate, cofactor and vitamin, lipid and nucleotide) and increased fat preference, offering a new field to reveal possible physiological progression markers (*N* = 30, *N* = 20) (Murley et al., 2020; Ahmed et al., 2021). However, for all suggested fluid biomarkers, e.g., NfL, sTREM2, GFAP, Orexin A as well as metabolic features, longitudinal observations are needed and highly recommended, before they can be evaluated in their potential to track disease progression.

## 4 Discussion

The purpose of this scoping review was to provide an overview of longitudinal studies in bvFTD and evaluate current assessment tools to monitor disease progression. The clinical markers of progression with most research evidence included FTD-specific rating scales, informant-rated multi-domain behavioral measures, comprehensive cognitive screener or composite scores, and few social cognition tools. The neuroimaging markers of progression with most research evidence included modalities detecting volumetric grey matter atrophy and white matter pathology, and to a lesser extent hypometabolism and hypoperfusion. Regarding fluid biomarkers, NfL was most researched and most valid, clearly showing significant decline over time. While more (extensive) longitudinal research and/or more sensitive markers of progression are advised, we propose a multimodal approach in bvFTD. To acknowledge the multi-dimensional heterogeneity, as found in behavior, cognition, neuroimaging features and biofluid levels, a combined set of progression markers is recommended, adjusted to genetic and sporadic variants.

**Figure 2:**
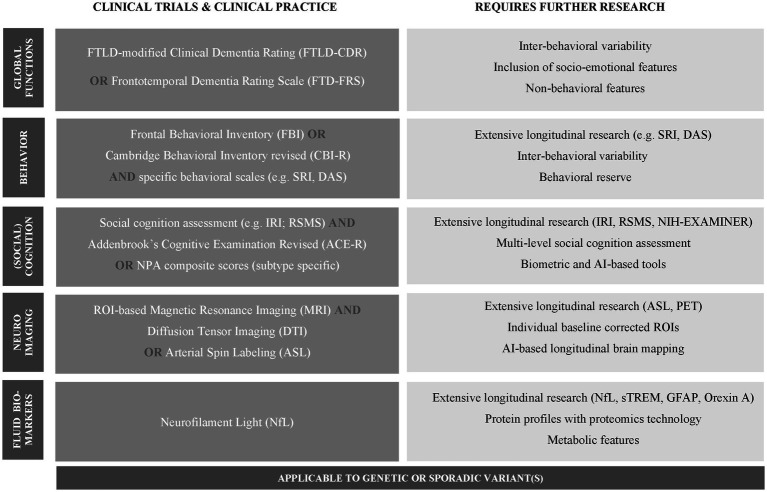
Central recommendations for a multi-modal approach and future research in bvFTD. ^*^SRI, Stereotypy Rating Inventory; DAS, Dimensional Apathy Scale; IRI, Interpersonal Reactivity Index; RSMS, Revised Self-Monitoring Scale; NPA, neuropsychological assessment; NIH-EXAMINER, Neurobehavioral Evaluation & Research; sTREM, soluble triggering receptor expressed on myeloid cells 2; GFAP, glial fibrillary acidic protein.

The central recommendations of this scoping review are listed in Figure 2. For future clinical trials, it is important to use outcome measures that are both easily administered and adequately detect clinically meaningful and biologically relevant changes in bvFTD. With regard to global functioning, the FTLD-CDR can be used for coarse staging, while the FTD-FRS offers a more sensitive measure for subtle changes and multiple domains. To anticipate on the complexity of behavioral change, i.e., heterogeneous profiles and inter-behavioral variability, the FBI or CBI-R are generally applicable due to their ability to aggregate the sum of behaviors, whereas separate specific scales (e.g., SRI or DAS) may be tailored to an individual’s baseline profile. Since clinical trials intend to intervene in early and intermediate stages, characterized by relatively diverse behavioral symptoms, behavioral inflection points should be taken into account. For instance, a crescendo-decrescendo pattern, including dominating apathy (measured with DAS or sub scores of FBI or CBI) in late stages, must be considered while interpreting, and ultimately modify, change over time. Regarding cognition, the ACE-R can be used as a brief and feasible screener, along with IRI and/or RSMS questionnaires assessing social cognitive changes. Given the fundamental and consistent role of socio-emotional deficits in the clinical phenotype of bvFTD, accurate social cognition assessment is prioritized over domain composite scores. When optimized, social cognition testing may provide easily administered and clinically meaningful measures, ideally related to specific biological changes and respecting individual (social) behavioral reserve. However, present social cognition tools require further longitudinal, preferably cross-cultural, validation and improved psychometrics to overcome floor effects. Targeted progress should focus on structured multi-level (social perception, interpretation and regulation) and multi-modal (informant-rated and patient-recorded/biometric) assessment, able to objectify gradual decline of social cognition. For neuroimaging, we suggest an approach on group level and individual level. On the group level, important ROIs for longitudinal change have been identified in frontal (incl. orbitofrontal), temporal, limbic (incl. anterior cingulate and insula) and striatal regions, next to genotype-specific GM atrophy patterns. In addition, WM disintegration patterns (DTI) and CBF changes (ASL) enable earlier and more sensitive detection than GM atrophy. Considering the need to capture individual variation, we suggest ROIs corrected for baseline atrophy patterns to follow individual-specific progression profiles. This may be used for individual monitoring in clinical practice, as well as averaged ROI-change in clinical trials. While upcoming techniques hold promise for gene and pathology-specific fluid biomarkers, current longitudinal studies indicate NfL as most potent progression marker in bvFTD. Importantly, rapid developments in technology point to novel digital biomarkers. While these are promising, at present, literature mostly involves cross-sectional studies in AD. Examples are speech-based artificial intelligence (AI) applications predicting cognitive decline (Fristed et al., 2022), biometric measures (e.g., skin conduction, pupillometry and eye-tracking patterns) reflecting social-emotional and/or linguistic deficits (Mendez et al., 2018; Singleton et al., 2022; El Haj et al., 2024), AI-based imaging algorithms for longitudinal brain mapping (Pérez-Millan et al., 2023), and proteomics technology detecting protein profiles (Katzeff et al., 2022).

Crucially, the majority of the large and leading studies on disease progression (of neuroimaging in particular) were predominantly performed in genetic cohorts of bvFTD (Staffaroni et al., 2019b, 2022). Genetic mutation carriers enable monitoring from pre-symptomatic to symptomatic stage, making them ideal for precise monitoring of disease progression from a preclinical stage. In contrast, sporadic cases are typically diagnosed years after symptom onset, resulting in more advanced stages at time of identification. The scarceness of longitudinal studies on the sporadic variant logically implies that current recommendations are based on fewer validation studies performed within sporadic bvFTD. Moreover, sporadic cases are frequently less defined and based on clinical diagnosis, rather than underlying pathology, affecting diagnostic certainty. However, since 70 % of bvFTD cases is non-genetic (Greaves and Rohrer, 2019), clearly this knowledge gap needs to be addressed. There is an urgent need for accurate phenotyping of sporadic bvFTD, identification and/or development of tailored outcome measures specific to sporadic cohorts, and proper stratification of patients in future clinical trials accordingly. This approach is essential for advancing our understanding of sporadic versus genetic bvFTD, and optimizing the effectiveness of therapeutic interventions across all variants of bvFTD.

Within this scoping review, there are multiple limitations to consider. A major challenge in interpretation and evaluation of findings was founded in the highly heterogeneous cohorts in bvFTD literature. Differences in patient populations (genetically undefined versus mutation-specific patients), follow-up time, study design (longitudinal follow-up versus cross-sectional associations with disease severity), and use of staging instruments less sensitive for bvFTD (e.g., traditional CDR) seriously complicated the comparative weighing of results. Due to this fact, meta-analysis was not possible, which would have further objectified and strengthened our findings. While the above-mentioned challenges are familiar in bvFTD literature, this scoping review also knows multiple strengths in the pursuit to overcome these obstacles. The systematic search of the vast literature (by means of extensive, inclusive search terms) was carried out in collaboration with a medical librarian, in accordance with evidence-based PRISMA standards, ensuring methodological rigor, and representing the status of literature in a complete and concise manner. The broad research question offered a comprehensive analysis of a wide spectrum of interdisciplinary domains, providing a relative comprehensive view of disease progression of value for future cohort development and trial design. Future research should focus on more extensive longitudinal follow-up for tool improvement and development, within large and well-defined cohorts, with regards to subtype, symptom onset and disease severity. Based on the present data we recommend to use a bvFTD-specific multi-modal battery to detect disease progression over time, including clinical, neuroimaging, and fluid biomarkers.

## Data availability statement

The original contributions presented in the study are included in the article/supplementary material, further inquiries can be directed to the corresponding authors.

## Author contributions

JF: Conceptualization, Data curation, Formal analysis, Investigation, Methodology, Project administration, Software, Validation, Visualization, Writing – original draft, Writing – review & editing. DP: Conceptualization, Data curation, Formal analysis, Investigation, Methodology, Project administration, Software, Validation, Visualization, Writing – original draft, Writing – review & editing. M-Pv: Writing – review & editing. SBo: Writing – review & editing. WH: Writing – review & editing. SBr: Writing – review & editing. LS: Data curation, Methodology, Writing – review & editing. SS: Writing – review & editing. WK: Writing – review & editing. MO: Writing – review & editing. HM: Writing – review & editing. CT: Writing – review & editing. EV: Supervision, Writing – review & editing. YP: Funding acquisition, Supervision, Writing – review & editing.

## References

[ref1] Agarwal S. Ahmed R. M. D’Mello M. Foxe D. Kaizik C. Kiernan M. C. . (2019). Predictors of survival and progression in behavioural variant frontotemporal dementia. Eur. J. Neurol. 26, 774–779. doi: 10.1111/ene.13887, PMID: 30565360

[ref2] Ahmed R. M. Tse N. Y. Chen Y. Henning E. Hodges J. R. Kiernan M. C. . (2021). Neural correlates of fat preference in frontotemporal dementia: translating insights from the obesity literature. Ann. Clin. Transl. Neurol. 8, 1318–1329. doi: 10.1002/acn3.51369, PMID: 33973740 PMC8164857

[ref3] Anderl-Straub S. Lausser L. Lombardi J. Uttner I. Fassbender K. Fliessbach K. . (2021). Predicting disease progression in behavioral variant frontotemporal dementia. Alzheimers Dement. 13:e12262. doi: 10.1002/dad2.12262, PMID: 35005196 PMC8719425

[ref4] Ash S. Nevler N. Phillips J. Irwin D. J. Mcmillan C. T. Rascovsky K. . (2019). A longitudinal study of speech production in primary progressive aphasia and behavioral variant frontotemporal dementia. Brain Lang. 194, 46–57. doi: 10.1016/j.bandl.2019.04.006, PMID: 31075725 PMC6656376

[ref5] Aw Y. Perrett D. Calder A. Sprengelmeyer R. Ekman P. (2002). Facial expressions of emotion: stimuli and tests (FEEST). Bury St Edmunds, England: Thames Valley Test Company (TVTC).

[ref6] Barker M. S. Manoochehri M. Rizer S. J. Appleby B. S. Brushaber D. Dev S. I. . (2021). Recognition memory and divergent cognitive profiles in prodromal genetic frontotemporal dementia. Cortex 139, 99–115. doi: 10.1016/j.cortex.2021.03.006, PMID: 33857770 PMC8119343

[ref7] Benussi A. Ashton N. J. Karikari T. K. Alberici A. Saraceno C. Ghidoni R. . (2021a). Prodromal frontotemporal dementia: clinical features and predictors of progression. Alzheimers Res. Ther. 13:188. doi: 10.1186/s13195-021-00932-2, PMID: 34782010 PMC8594126

[ref8] Benussi A. Karikari T. K. Ashton N. Gazzina S. Premi E. Benussi L. . (2020). Diagnostic and prognostic value of serum NfL and p-Tau_181_ in frontotemporal lobar degeneration. J. Neurol. Neurosurg. Psychiatry 91, 960–967. doi: 10.1136/jnnp-2020-323487 32611664

[ref9] Benussi A. Libri I. Premi E. Alberici A. Cantoni V. Gadola Y. . (2022). Differences and similarities between familial and sporadic frontotemporal dementia: an Italian single-center cohort study. Alzheimers Dement. 8:e12326. doi: 10.1002/trc2.12326, PMID: 35898667 PMC9310192

[ref10] Benussi A. Premi E. Gazzina S. Brattini C. Bonomi E. Alberici A. . (2021b). Progression of behavioral disturbances and neuropsychiatric symptoms in patients with genetic frontotemporal dementia. JAMA Netw. Open 4:e2030194. doi: 10.1001/jamanetworkopen.2020.30194, PMID: 33404617 PMC7788468

[ref11] Bickart K. C. Brickhouse M. Negreira A. Sapolsky D. Barrett L. F. Dickerson B. C. (2014). Atrophy in distinct corticolimbic networks in frontotemporal dementia relates to social impairments measured using the social impairment rating scale. J. Neurol. Neurosurg. Psychiatry 85, 438–448. doi: 10.1136/jnnp-2012-304656, PMID: 24133285 PMC4315506

[ref12] Binney R. J. Pankov A. Marx G. He X. Mckenna F. Staffaroni A. M. . (2017). Data-driven regions of interest for longitudinal change in three variants of frontotemporal lobar degeneration. Brain Behav. 7:e00675. doi: 10.1002/brb3.675, PMID: 28413716 PMC5390848

[ref13] Blair M. Marczinski C. A. Davis-Faroque N. Kertesz A. (2007). A longitudinal study of language decline in Alzheimer’s disease and frontotemporal dementia. J. Int. Neuropsychol. Soc. 13, 237–245. doi: 10.1017/S1355617707070269, PMID: 17286881

[ref14] Borges L. G. Rademaker A. W. Bigio E. H. Mesulam M. M. Weintraub S. (2019). Apathy and disinhibition related to neuropathology in amnestic versus behavioral dementias. Am. J. Alzheimers Dis. Other Dement. 34, 337–343. doi: 10.1177/1533317519853466, PMID: 31170813 PMC7256964

[ref15] Borroni B. Agosti C. Premi E. Cerini C. Cosseddu M. Paghera B. . (2010). The FTLD-modified Clinical Dementia Rating scale is a reliable tool for defining disease severity in frontotemporal lobar degeneration: evidence from a brain SPECT study. Eur. J. Neurol. 17, 703–707. doi: 10.1111/j.1468-1331.2009.02911.x, PMID: 20050899

[ref16] Boutoleau-Bretonniere C. Lebouvier T. Volteau C. Jaulin P. Lacomblez L. Damier P. . (2012). Prospective evaluation of behavioral scales in the behavioral variant of frontotemporal dementia. Dement. Geriatr. Cogn. Disord. 34, 75–82. doi: 10.1159/000341784, PMID: 22922703

[ref17] Brambati S. M. Renda N. C. Rankin K. P. Rosen H. J. Seeley W. W. Ashburner J. . (2007). A tensor based morphometry study of longitudinal gray matter contraction in FTD. NeuroImage 35, 998–1003. doi: 10.1016/j.neuroimage.2007.01.028, PMID: 17350290 PMC2443736

[ref18] Bramer W. M. Giustini D. De Jonge G. B. Holland L. Bekhuis T. (2016). De-duplication of database search results for systematic reviews in endnote. J. Med. Libr. Assoc. 104, 240–243. doi: 10.3163/1536-5050.104.3.014, PMID: 27366130 PMC4915647

[ref19] Carnemolla S. E. Kumfor F. Liang C. T. Foxe D. Ahmed R. M. Piguet O. (2022). Olfactory bulb integrity in frontotemporal dementia and Alzheimer’s disease. J. Alzheimers Dis. 89, 51–66. doi: 10.3233/JAD-220080, PMID: 35848020

[ref20] Chen Q. Kantarci K. (2020). Imaging biomarkers for neurodegeneration in Presymptomatic familial frontotemporal lobar degeneration. Front. Neurol. 11:80. doi: 10.3389/fneur.2020.00080, PMID: 32184751 PMC7058699

[ref21] Chow T. W. Fridhandler J. D. Binns M. A. Lee A. Merrilees J. Rosen H. J. . (2012). Trajectories of behavioral disturbance in dementia. J. Alzheimers Dis. 31, 143–149. doi: 10.3233/JAD-2012-111916, PMID: 22531424 PMC4309273

[ref22] Cosseddu M. Benussi A. Gazzina S. Alberici A. Dell’era V. Manes M. . (2019). Progression of behavioural disturbances across frontotemporal dementia: a longitudinal observational study. Eur. J. Neurol. 27, 265–272. doi: 10.1111/ene.14071 31448481

[ref23] Cummings J. L. Mega M. Gray K. Rosenberg-Thompson S. Carusi D. A. Gornbein J. (1994). The neuropsychiatric inventory: comprehensive assessment of psychopathology in dementia. Neurology 44:2308. doi: 10.1212/WNL.44.12.2308 7991117

[ref24] Da Silva T. B. L. Ordonez T. N. Bregola A. G. Bahia V. S. Cecchini M. A. Guimarães H. C. . (2021). Neuropsychiatric symptoms in behavioral variant frontotemporal dementia and Alzheimer’s disease: a 12-month follow-up study. Front. Neurol. 12:728108. doi: 10.3389/fneur.2021.728108, PMID: 34659093 PMC8515178

[ref25] Davis M. (1980). A multidimensional approach to individual differences in empathy. JSAS Catalog Sel. Doc. Psychol 10:85,

[ref26] Davis M. (1983). Measuring individual differences in empathy: evidence for a multidimensional approach. J. Pers. Soc. Psychol. 44, 113–126. doi: 10.1037/0022-3514.44.1.113

[ref27] Day G. S. Farb N. A. Tang-Wai D. F. Masellis M. Black S. E. Freedman M. . (2013). Salience network resting-state activity: prediction of frontotemporal dementia progression. JAMA Neurol. 70, 1249–1253. doi: 10.1001/jamaneurol.2013.3258, PMID: 23959214 PMC12893382

[ref28] De Boer S. C. M. Gossink F. Krudop W. Vijverberg E. Schouws S. Reus L. M. . (2023). Diagnostic instability over time in the late-onset frontal lobe syndrome: when can we say it’s FTD? Am. J. Geriatr. Psychiatry 31, 679–690. doi: 10.1016/j.jagp.2023.02.006, PMID: 37028983

[ref29] Devenney E. Bartley L. Hoon C. O’Callaghan C. Kumfor F. Hornberger M. . (2015). Progression in behavioral variant frontotemporal dementia: a longitudinal study. JAMA Neurol. 72, 1501–1509. doi: 10.1001/jamaneurol.2015.2061 26501846

[ref30] Devenney E. Hornberger M. Irish M. Mioshi E. Burrell J. Tan R. . (2014). Frontotemporal dementia associated with the C9ORF72 mutation: a unique clinical profile. JAMA Neurol. 71, 331–339. doi: 10.1001/jamaneurol.2013.6002 24445580

[ref31] Devenney E. Swinn T. Mioshi E. Hornberger M. Dawson K. E. Mead S. . (2018). The behavioural variant frontotemporal dementia phenocopy syndrome is a distinct entity—evidence from a longitudinal study. BMC Neurol. 18:56. doi: 10.1186/s12883-018-1060-1, PMID: 29704893 PMC5923010

[ref32] Diehl-Schmid J. Bornschein S. Pohl C. Forstl H. Kurz A. Jahn T. (2011). Cognitive decline in the behavioral variant of frontotemporal dementia. Int. Psychogeriatr. 23, 230–237. doi: 10.1017/S104161021000164X 20836915

[ref33] Diehl-Schmid J. Grimmer T. Drzezga A. Bornschein S. Riemenschneider M. Forstl H. . (2007). Decline of cerebral glucose metabolism in frontotemporal dementia: a longitudinal 18F-FDG-PET-study. Neurobiol. Aging 28, 42–50. doi: 10.1016/j.neurobiolaging.2005.11.002, PMID: 16448722

[ref34] Diehl-Schmid J. Pohl C. Perneczky R. Forstl H. Kurz A. (2006). Behavioral disturbances in the course of frontotemporal dementia. Dement. Geriatr. Cogn. Disord. 22, 352–357. doi: 10.1159/000095625 16954691

[ref35] Dodich A. Carli G. Cerami C. Iannaccone S. Magnani G. Perani D. (2018). Social and cognitive control skills in long-life occupation activities modulate the brain reserve in the behavioural variant of frontotemporal dementia. Cortex 99, 311–318. doi: 10.1016/j.cortex.2017.12.006 29328983

[ref36] Dopper E. G. Chalos V. Ghariq E. Den Heijer T. Hafkemeijer A. Jiskoot L. C. . (2016). Cerebral blood flow in presymptomatic MAPT and GRN mutation carriers: a longitudinal arterial spin labeling study. NeuroImage Clin. 12, 460–465. doi: 10.1016/j.nicl.2016.08.001, PMID: 27625986 PMC5011170

[ref37] Dubois B. Slachevsky A. Litvan I. Pillon B. (2000). The FAB: a frontal assessment battery at bedside. Neurology 55, 1621–1626. doi: 10.1212/WNL.55.11.1621 11113214

[ref38] El Haj M. Kapogiannis D. Boutoleau-Bretonnière C. (2024). Pupil size shows diminished increases on verbal fluency tasks in patients with behavioral-variant-frontotemporal dementia. J. Neurolinguistics 69:101164. doi: 10.1016/j.jneuroling.2023.101164

[ref39] Elahi F. M. Marx G. Cobigo Y. Staffaroni A. M. Kornak J. Tosun D. . (2017). Longitudinal White matter change in frontotemporal dementia subtypes and sporadic late onset Alzheimer’s disease. NeuroImage Clin. 16, 595–603. doi: 10.1016/j.nicl.2017.09.007, PMID: 28975068 PMC5614750

[ref40] Feneberg E. Steinacker P. Volk A. E. Weishaupt J. H. Wollmer M. A. Boxer A. . (2016). Progranulin as a candidate biomarker for therapeutic trial in patients with ALS and FTLD. J. Neural Transm. 123, 289–296. doi: 10.1007/s00702-015-1486-1, PMID: 26659729

[ref41] Fieldhouse J. L. P. Gossink F. T. Feenstra T. C. De Boer S. C. M. Lemstra A. W. Prins N. D. . (2021). Clinical phenotypes of behavioral variant frontotemporal dementia by age at onset. J. Alzheimers Dis. 82, 381–390. doi: 10.3233/JAD-210179, PMID: 34024833 PMC8293634

[ref42] Floeter M. K. Bageac D. Danielian L. E. Braun L. E. Traynor B. J. Kwan J. Y. (2016). Longitudinal imaging in C9ORF72 mutation carriers: relationship to phenotype. NeuroImage Clin. 12, 1035–1043. doi: 10.1016/j.nicl.2016.10.014, PMID: 27995069 PMC5153604

[ref43] Floeter M. K. Danielian L. E. Braun L. E. Wu T. (2018). Longitudinal diffusion imaging across the C9ORF72 clinical spectrum. J. Neurol. Neurosurg. Psychiatry 89, 53–60. doi: 10.1136/jnnp-2017-316799, PMID: 29054917 PMC6454927

[ref44] Floeter M. K. Traynor B. J. Farren J. Braun L. E. Tierney M. Wiggs E. A. . (2017). Disease progression in C9ORF72 mutation carriers. Neurology 89, 234–241. doi: 10.1212/WNL.0000000000004115, PMID: 28615433 PMC5513817

[ref45] Foiani M. S. Woollacott I. O. Heller C. Bocchetta M. Heslegrave A. Dick K. M. . (2018). Plasma tau is increased in frontotemporal dementia. J. Neurol. Neurosurg. Psychiatry 89, 804–807. doi: 10.1136/jnnp-2017-317260, PMID: 29440230 PMC6204947

[ref46] Folstein M. F. Folstein S. E. Mchugh P. R. (1975). “Mini-mental state”. A practical method for grading the cognitive state of patients for the clinician. J. Psychiatr. Res. 12, 189–198. doi: 10.1016/0022-3956(75)90026-6 1202204

[ref47] Foster P. H. Russell L. L. Peakman G. Convery R. S. Bouzigues A. Greaves C. V. . (2022). Examining empathy deficits across familial forms of frontotemporal dementia within the GENFI cohort. Cortex 150, 12–28. doi: 10.1016/j.cortex.2022.01.012, PMID: 35325762 PMC9067453

[ref48] Frings L. Mader I. Landwehrmeyer B. G. Weiller C. Hull M. Huppertz H. J. (2012). Quantifying change in individual subjects affected by frontotemporal lobar degeneration using automated longitudinal MRI volumetry. Hum. Brain Mapp. 33, 1526–1535. doi: 10.1002/hbm.21304 21618662 PMC6869947

[ref49] Frings L. Yew B. Flanagan E. Lam B. Y. Hull M. Huppertz H. J. . (2014). Longitudinal grey and white matter changes in frontotemporal dementia and Alzheimer’s disease. PLoS One 9:e90814. doi: 10.1371/journal.pone.0090814, PMID: 24595028 PMC3940927

[ref50] Fristed E. Skirrow C. Meszaros M. Lenain R. Meepegama U. Cappa S. . (2022). A remote speech-based AI system to screen for early Alzheimer’s disease via smartphones. Alzheimers Dement. 14:e12366. doi: 10.1002/dad2.12366, PMID: 36348974 PMC9632864

[ref51] Garcin B. Lillo P. Hornberger M. Piguet O. Dawson K. Nestor P. J. . (2009). Determinants of survival in behavioral variant frontotemporal dementia. Neurology 73, 1656–1661. doi: 10.1212/WNL.0b013e3181c1dee7, PMID: 19917988 PMC2881857

[ref52] Gélinas I. Gauthier L. Mcintyre M. Gauthier S. (1999). Development of a functional measure for persons with Alzheimer’s disease: the disability assessment for dementia. Am. J. Occup. Ther. 53, 471–481. doi: 10.5014/ajot.53.5.471 10500855

[ref53] Gendron T. F. Heckman M. G. White L. J. Veire A. M. Pedraza O. Burch A. R. . (2022). Comprehensive cross-sectional and longitudinal analyses of plasma neurofilament light across FTD spectrum disorders. Cell Rep. Med. 3:100607. doi: 10.1016/j.xcrm.2022.100607, PMID: 35492244 PMC9044101

[ref54] Giebel C. M. Knopman D. Mioshi E. Khondoker M. (2021). Distinguishing frontotemporal dementia from Alzheimer disease through everyday function profiles: trajectories of change. J. Geriatr. Psychiatry Neurol. 34, 66–75. doi: 10.1177/0891988720901791, PMID: 32054376 PMC7423644

[ref55] Gordon E. Bocchetta M. Nicholas J. Cash D. M. Rohrer J. D. (2021). A comparison of automated atrophy measures across the frontotemporal dementia spectrum: implications for trials. NeuroImage Clin. 32:102842. doi: 10.1016/j.nicl.2021.102842, PMID: 34626889 PMC8503665

[ref56] Gordon E. Rohrer J. D. Kim L. G. Omar R. Rossor M. N. Fox N. C. . (2010). Measuring disease progression in frontotemporal lobar degeneration: a clinical and MRI study. Neurology 74, 666–673. doi: 10.1212/WNL.0b013e3181d1a879, PMID: 20177120 PMC2830919

[ref57] Gossink F. T. Vijverberg E. Krudop W. Scheltens P. Stek M. L. Pijnenburg Y. A. L. . (2019). Predicting progression in the late onset frontal lobe syndrome. Int. Psychogeriatr. 31, 743–748. doi: 10.1017/S1041610218001242, PMID: 30362933

[ref58] Greaves C. V. Rohrer J. D. (2019). An update on genetic frontotemporal dementia. J. Neurol. 266, 2075–2086. doi: 10.1007/s00415-019-09363-4, PMID: 31119452 PMC6647117

[ref59] Grossman M. Xie S. X. Libon D. J. Wang X. Massimo L. Moore P. . (2008). Longitudinal decline in autopsy-defined frontotemporal lobar degeneration. Neurology 70, 2036–2045. doi: 10.1212/01.wnl.0000303816.25065.bc, PMID: 18420483 PMC2736475

[ref60] Hogan D. B. Jetté N. Fiest K. M. Roberts J. I. Pearson D. Smith E. E. . (2016). The prevalence and incidence of frontotemporal dementia: a systematic review. Can. J. Neurol. Sci. 43, S96–S109. doi: 10.1017/cjn.2016.25 27307130

[ref61] Hornberger M. Piguet O. Kipps C. Hodges J. R. (2008). Executive function in progressive and nonprogressive behavioral variant frontotemporal dementia. Neurology 71, 1481–1488. doi: 10.1212/01.wnl.0000334299.72023.c8, PMID: 18981369

[ref62] Hornberger M. Shelley B. P. Kipps C. M. Piguet O. Hodges J. R. (2009). Can progressive and non-progressive behavioural variant frontotemporal dementia be distinguished at presentation? J. Neurol. Neurosurg. Psychiatry 80, 591–593. doi: 10.1136/jnnp.2008.163873, PMID: 19228667

[ref63] Hughes L. E. Rowe J. B. (2013). The impact of neurodegeneration on network connectivity: a study of change detection in frontotemporal dementia. J. Cogn. Neurosci. 25, 802–813. doi: 10.1162/jocn_a_00356, PMID: 23469882 PMC3708294

[ref64] Huston J. 3rd Murphy M. C. Boeve B. F. Fattahi N. Arani A. Glaser K. J. . (2016). Magnetic resonance elastography of frontotemporal dementia. J. Magn. Reson. Imaging 43, 474–478. doi: 10.1002/jmri.24977, PMID: 26130216 PMC4696917

[ref65] Hutchings R. Palermo R. Bruggemann J. Hodges J. R. Piguet O. Kumfor F. (2018). Looking but not seeing: increased eye fixations in behavioural-variant frontotemporal dementia. Cortex 103, 71–81. doi: 10.1016/j.cortex.2018.02.011 29573594

[ref66] Huynh K. Piguet O. Kwok J. Dobson-Stone C. Halliday G. M. Hodges J. R. . (2021). Clinical and biological correlates of white matter hyperintensities in patients with behavioral-variant frontotemporal dementia and Alzheimer disease. Neurology 96, e1743–e1754. doi: 10.1212/WNL.0000000000011638, PMID: 33597290

[ref67] Illán-Gala I. Casaletto K. B. Borrego-Écija S. Arenaza-Urquijo E. M. Wolf A. Cobigo Y. . (2021a). Sex differences in the behavioral variant of frontotemporal dementia: a new window to executive and behavioral reserve. Alzheimers Dement. 17, 1329–1341. doi: 10.1002/alz.12299, PMID: 33590953 PMC8364861

[ref68] Illán-Gala I. Falgàs N. Friedberg A. Castro-Suárez S. Keret O. Rogers N. . (2021b). Diagnostic utility of measuring cerebral atrophy in the behavioral variant of frontotemporal dementia and association with clinical deterioration. JAMA Netw. Open 4:e211290. doi: 10.1001/jamanetworkopen.2021.1290, PMID: 33704477 PMC7953307

[ref69] Irish M. Graham A. Graham K. S. Hodges J. R. Hornberger M. (2012). Differential impairment of source memory in progressive versus non-progressive behavioral variant frontotemporal dementia. Arch. Clin. Neuropsychol. 27, 338–347. doi: 10.1093/arclin/acs033, PMID: 22414677

[ref70] Irish M. Landin-Romero R. Mothakunnel A. Ramanan S. Hsieh S. Hodges J. R. . (2018). Evolution of autobiographical memory impairments in Alzheimer’s disease and frontotemporal dementia—a longitudinal neuroimaging study. Neuropsychologia 110, 14–25. doi: 10.1016/j.neuropsychologia.2017.03.014, PMID: 28288787

[ref71] Jiskoot L. C. Poos J. M. Vollebergh M. E. Franzen S. Van Hemmen J. Papma J. M. . (2021). Emotion recognition of morphed facial expressions in presymptomatic and symptomatic frontotemporal dementia, and Alzheimer’s dementia. J. Neurol. 268, 102–113. doi: 10.1007/s00415-020-10096-y, PMID: 32728945 PMC7815624

[ref72] Josephs K. A. Jr. Whitwell J. L. Weigand S. D. Senjem M. L. Boeve B. F. Knopman D. S. . (2011). Predicting functional decline in behavioural variant frontotemporal dementia. Brain 134, 432–448. doi: 10.1093/brain/awq348, PMID: 21252111 PMC3030765

[ref73] Joshi A. Mendez M. Kaiser N. Jimenez E. Mather M. (2014). Skin conductance levels may reflect emotional blunting in behavioral variant frontotemporal dementia. J. Neuropsychiatry Clin. Neurosci. 26, 227–232. doi: 10.1176/appi.neuropsych.12110332, PMID: 25093763

[ref74] Kassubek J. Muller H. P. Del Tredici K. Hornberger M. Schroeter M. L. Muller K. . (2018). Longitudinal diffusion tensor imaging resembles patterns of pathology progression in behavioral variant frontotemporal dementia (BVFTD). Front. Aging Neurosci. 10:47. doi: 10.3389/fnagi.2018.00047, PMID: 29559904 PMC5845670

[ref75] Katz S. Ford A. B. Moskowitz R. W. Jackson B. A. Jaffe M. W. (1963). Studies of illness in the aged. The index of ADL: a standardized measure of biological and psychosocial function. JAMA 185, 914–919. doi: 10.1001/jama.1963.03060120024016 14044222

[ref76] Katzeff J. S. Bright F. Phan K. Kril J. J. Ittner L. M. Kassiou M. . (2022). Biomarker discovery and development for frontotemporal dementia and amyotrophic lateral sclerosis. Brain 145, 1598–1609. doi: 10.1093/brain/awac077, PMID: 35202463 PMC9166557

[ref77] Kawakami I. Arai T. Shinagawa S. Niizato K. Oshima K. Ikeda M. (2021). Distinct early symptoms in neuropathologically proven frontotemporal lobar degeneration. Int. J. Geriatr. Psychiatry 36, 38–45. doi: 10.1002/gps.5387, PMID: 32748432

[ref78] Kazui H. Yoshiyama K. Kanemoto H. Suzuki Y. Sato S. Hashimoto M. . (2016). Differences of behavioral and psychological symptoms of dementia in disease severity in four major dementias. PLoS One 11:e0161092. doi: 10.1371/journal.pone.0161092, PMID: 27536962 PMC4990196

[ref79] Kertesz A. Davidson W. Fox H. (1997). Frontal behavioral inventory: diagnostic criteria for frontal lobe dementi. Can. J. Neurol. Sci. 24, 29–36. doi: 10.1017/S0317167100021053 9043744

[ref80] Kessels R. P. Gerritsen L. Montagne B. Ackl N. Diehl J. Danek A. (2007). Recognition of facial expressions of different emotional intensities in patients with frontotemporal lobar degeneration. Behav. Neurol. 18, 31–36. doi: 10.1155/2007/868431, PMID: 17297217 PMC5469962

[ref81] Kim S. H. Kim Y. J. Lee B. H. Lee P. Park J. H. Seo S. W. . (2022). Behavioral reserve in behavioral variant frontotemporal dementia. Front. Aging Neurosci. 14:875589. doi: 10.3389/fnagi.2022.875589, PMID: 35795232 PMC9252599

[ref82] Kinney N. G. Bove J. Phillips J. S. Cousins K. A. Q. Olm C. A. Wakeman D. G. . (2021). Social and leisure activity are associated with attenuated cortical loss in behavioral variant frontotemporal degeneration. NeuroImage Clin. 30:102629. doi: 10.1016/j.nicl.2021.102629, PMID: 33770546 PMC8024767

[ref83] Knopman D. S. Jack C. R. Jr. Kramer J. H. Boeve B. F. Caselli R. J. Graff-Radford N. R. . (2009). Brain and ventricular volumetric changes in frontotemporal lobar degeneration over 1 year. Neurology 72, 1843–1849. doi: 10.1212/WNL.0b013e3181a71236, PMID: 19470967 PMC2690986

[ref84] Knopman D. S. Kramer J. H. Boeve B. F. Caselli R. J. Graff-Radford N. R. Mendez M. F. . (2008). Development of methodology for conducting clinical trials in frontotemporal lobar degeneration. Brain 131, 2957–2968. doi: 10.1093/brain/awn234, PMID: 18829698 PMC2725027

[ref85] Knopman D. S. Weintraub S. Pankratz V. S. (2011). Language and behavior domains enhance the value of the clinical dementia rating scale. Alzheimers Dement. 7, 293–299. doi: 10.1016/j.jalz.2010.12.006, PMID: 21575870 PMC3096831

[ref86] Kril J. J. Macdonald V. Patel S. Png F. Halliday G. M. (2005). Distribution of brain atrophy in behavioral variant frontotemporal dementia. J. Neurol. Sci. 232, 83–90. doi: 10.1016/j.jns.2005.02.003 15850587

[ref87] Krudop W. A. Dols A. Kerssens C. J. Eikelenboom P. Prins N. D. Möller C. . (2017). The pitfall of behavioral variant frontotemporal dementia mimics despite multidisciplinary application of the Ftdc criteria. J. Alzheimers Dis. 60, 959–975. doi: 10.3233/JAD-170608 28984605

[ref88] Kumfor F. Irish M. Leyton C. Miller L. Lah S. Devenney E. . (2014). Tracking the progression of social cognition in neurodegenerative disorders. J. Neurol. Neurosurg. Psychiatry 85, 1076–1083. doi: 10.1136/jnnp-2013-307098, PMID: 24569686

[ref89] Laganà V. Bruno F. Altomari N. Bruni G. Smirne N. Curcio S. . (2022). Neuropsychiatric or behavioral and psychological symptoms of dementia (BPSD): focus on prevalence and natural history in Alzheimer’s disease and frontotemporal dementia. Front. Neurol. 13:832199. doi: 10.3389/fneur.2022.832199 35812082 PMC9263122

[ref90] Lam B. Y. Halliday G. M. Irish M. Hodges J. R. Piguet O. (2014). Longitudinal white matter changes in frontotemporal dementia subtypes. Hum. Brain Mapp. 35, 3547–3557. doi: 10.1002/hbm.22420, PMID: 25050433 PMC6869363

[ref91] Landin-Romero R. Kumfor F. Leyton C. E. Irish M. Hodges J. R. Piguet O. (2017). Disease-specific patterns of cortical and subcortical degeneration in a longitudinal study of Alzheimer’s disease and behavioural-variant frontotemporal dementia. NeuroImage 151, 72–80. doi: 10.1016/j.neuroimage.2016.03.032, PMID: 27012504

[ref92] Lansdall C. J. Coyle-Gilchrist I. T. S. Vazquez Rodriguez P. Wilcox A. Wehmann E. Robbins T. W. . (2019). Prognostic importance of apathy in syndromes associated with frontotemporal lobar degeneration. Neurology 92, e1547–e1557. doi: 10.1212/WNL.0000000000007249, PMID: 30842292 PMC6448451

[ref93] Lavenu I. Pasquier F. (2005). Perception of emotion on faces in frontotemporal dementia and Alzheimer’s disease: a longitudinal study. Dement. Geriatr. Cogn. Disord. 19, 37–41. doi: 10.1159/000080969, PMID: 15383744

[ref94] Lawton M. P. Brody E. M. (1969). Assessment of older people: self-maintaining and instrumental activities of daily living. Gerontologist 9, 179–186. doi: 10.1093/geront/9.3_Part_1.179, PMID: 5349366

[ref95] Le Ber I. Guedj E. Gabelle A. Verpillat P. Volteau M. Thomas-Anterion C. . (2006). Demographic, neurological and behavioural characteristics and brain perfusion SPECT in frontal variant of frontotemporal dementia. Brain 129, 3051–3065. doi: 10.1093/brain/awl288, PMID: 17071924

[ref96] Leroy M. Bertoux M. Skrobala E. Mode E. Adnet-Bonte C. Le Ber I. . (2021). Characteristics and progression of patients with frontotemporal dementia in a regional memory clinic network. Alzheimers Res. Ther. 13:19. doi: 10.1186/s13195-020-00753-9, PMID: 33419472 PMC7796569

[ref97] Lima-Silva T. B. Mioshi E. Bahia V. S. Cecchini M. A. Cassimiro L. Guimarães H. C. . (2021). Disease progression in frontotemporal dementia and Alzheimer disease: the contribution of staging scales. J. Geriatr. Psychiatry Neurol. 34, 397–404. doi: 10.1177/0891988720944239, PMID: 32762416

[ref98] Linds A. B. Kirstein A. B. Freedman M. Verhoeff N. P. Wolf U. Chow T. W. (2015). Trajectories of behavioural disturbances across dementia types. Can. J. Neurol. Sci. 42, 389–394. doi: 10.1017/cjn.2015.266, PMID: 26329453

[ref99] Ljubenkov P. A. Staffaroni A. M. Rojas J. C. Allen I. E. Wang P. Heuer H. . (2018). Cerebrospinal fluid biomarkers predict frontotemporal dementia trajectory. Ann. Clin. Transl. Neurol. 5, 1250–1263. doi: 10.1002/acn3.643, PMID: 30349860 PMC6186942

[ref100] Macfarlane M. D. Jakabek D. Walterfang M. Vestberg S. Velakoulis D. Wilkes F. A. . (2015). Striatal atrophy in the behavioural variant of frontotemporal dementia: correlation with diagnosis, negative symptoms and disease severity. PLoS One 10:e0129692. doi: 10.1371/journal.pone.0129692, PMID: 26075893 PMC4468218

[ref101] Mahoney C. J. Simpson I. J. Nicholas J. M. Fletcher P. D. Downey L. E. Golden H. L. . (2015). Longitudinal diffusion tensor imaging in frontotemporal dementia. Ann. Neurol. 77, 33–46. doi: 10.1002/ana.24296, PMID: 25363208 PMC4305215

[ref102] Maiovis P. Ioannidis P. Gerasimou G. Gotzamani-Psarrakou A. Karacostas D. (2017). Frontotemporal lobar degeneration-modified clinical dementia rating (FTLD-CDR) scale and frontotemporal dementia rating scale (FRS) correlation with regional brain perfusion in a series of FTLD patients. J. Neuropsychiatry Clin. Neurosci. 29, 26–30. doi: 10.1176/appi.neuropsych.16020034, PMID: 27417072

[ref103] Maiovis P. Ioannidis P. Gerasimou G. Gotzamani-Psarrakou A. Karacostas D. (2018). Cognitive reserve hypothesis in frontotemporal dementia: evidence from a brain SPECT study in a series of Greek frontotemporal dementia patients. Neurodegener. Dis. 18, 69–73. doi: 10.1159/000486621, PMID: 29514157

[ref104] Malpetti M. Holland N. Jones P. S. Ye R. Cope T. E. Fryer T. D. . (2021). Synaptic density in carriers of C9orf72 mutations: a [^11^C]UCB-J PET study. Ann. Clin. Transl. Neurol. 8, 1515–1523. doi: 10.1002/acn3.51407, PMID: 34133849 PMC8283163

[ref105] Malpetti M. Simon Jones P. Cope T. E. Holland N. Naessens M. Rouse M. A. . (2022). Synaptic loss in behavioural variant frontotemporal dementia revealed by [^11^C]UCB-J PET. Cambridge, United Kingdom: Department Of Clinical Neurosciences, University Of Cambridge, Herchel Smith Building, Cambridge Biomedical Campus.

[ref106] Manera A. L. Dadar M. Collins D. L. Ducharme S. (2019). Deformation based morphometry study of longitudinal MRI changes in behavioral variant frontotemporal dementia. NeuroImage Clin. 24:102079. doi: 10.1016/j.nicl.2019.102079, PMID: 31795051 PMC6879994

[ref107] Marczinski C. A. Davidson W. Kertesz A. (2004). A longitudinal study of behavior in frontotemporal dementia and primary progressive aphasia. Cogn. Behav. Neurol. 17, 185–190, PMID: 15622012

[ref108] Massimo L. Rennert L. Xie S. X. Olm C. Bove J. Van Deerlin V. . (2021). Common genetic variation is associated with longitudinal decline and network features in behavioral variant frontotemporal degeneration. Neurobiol. Aging 108, 16–23. doi: 10.1016/j.neurobiolaging.2021.07.018, PMID: 34474300 PMC8616801

[ref109] Massimo L. Xie S. X. Rennert L. Fick D. M. Halpin A. Placek K. . (2019). Occupational attainment influences longitudinal decline in behavioral variant frontotemporal degeneration. Brain Imaging Behav. 13, 293–301. doi: 10.1007/s11682-018-9852-x, PMID: 29542053 PMC6521965

[ref110] Mcdonald S. Flanagan S. Rollins J. Kinch J. (2003). TASIT: a new clinical tool for assessing social perception after traumatic brain injury. J. Head Trauma Rehabil. 18, 219–238. doi: 10.1097/00001199-200305000-00001 12802165

[ref111] Meeter L. H. Kaat L. D. Rohrer J. D. Van Swieten J. C. (2017). Imaging and fluid biomarkers in frontotemporal dementia. Nat. Rev. Neurol. 13, 406–419. doi: 10.1038/nrneurol.2017.75 28621768

[ref112] Meeter L. H. H. Vijverberg E. G. Del Campo M. Rozemuller A. J. M. Donker Kaat L. De Jong F. J. . (2018). Clinical value of neurofilament and phospho-tau/tau ratio in the frontotemporal dementia spectrum. Neurology 90, e1231–e1239. doi: 10.1212/WNL.0000000000005261, PMID: 29514947 PMC5890612

[ref113] Mendez M. F. Fong S. S. Ashla M. M. Jimenez E. E. Carr A. R. (2018). Skin conduction levels differentiate frontotemporal dementia from Alzheimer’s disease. J. Neuropsychiatry Clin. Neurosci. 30, 208–213. doi: 10.1176/appi.neuropsych.17080168, PMID: 29621927 PMC6081247

[ref114] Mendez M. F. Shapira J. S. (2011). Loss of emotional insight in behavioral variant frontotemporal dementia or “frontal anosodiaphoria”. Conscious. Cogn. 20, 1690–1696. doi: 10.1016/j.concog.2011.09.005, PMID: 21959203 PMC3199289

[ref115] Midorikawa A. Leyton C. E. Foxe D. Landin-Romero R. Hodges J. R. Piguet O. (2016). All is not lost: positive behaviors in Alzheimer’s disease and behavioral-variant frontotemporal dementia with disease severity. J. Alzheimers Dis. 54, 549–558. doi: 10.3233/JAD-160440, PMID: 27472884 PMC5026134

[ref116] Mioshi E. Dawson K. Mitchell J. Arnold R. Hodges J. R. (2006). The Addenbrooke’s cognitive examination revised (ACE-R): a brief cognitive test battery for dementia screening. Int. J. Geriatr. Psychiatry 21, 1078–1085. doi: 10.1002/gps.1610, PMID: 16977673

[ref117] Mioshi E. Flanagan E. Knopman D. (2017). Detecting clinical change with the CDR-FTLD: differences between FTLD and AD dementia. Int. J. Geriatr. Psychiatry 32, 977–982. doi: 10.1002/gps.4556, PMID: 27464599 PMC5274594

[ref118] Mioshi E. Hodges J. R. (2009). Rate of change of functional abilities in frontotemporal dementia. Dement. Geriatr. Cogn. Disord. 28, 419–426. doi: 10.1159/000255652, PMID: 19907178

[ref119] Mioshi E. Hsieh S. Savage S. Hornberger M. Hodges J. R. (2010). Clinical staging and disease progression in frontotemporal dementia. Neurology 74, 1591–1597. doi: 10.1212/WNL.0b013e3181e04070 20479357

[ref120] Morris J. C. (1993). The clinical dementia rating (CDR): current version and scoring rules. Neurology 43, 2412–2414. doi: 10.1212/WNL.43.11.2412-a, PMID: 8232972

[ref121] Murley A. G. Jones P. S. Coyle Gilchrist I. Bowns L. Wiggins J. Tsvetanov K. A. . (2020). Metabolomic changes associated with frontotemporal lobar degeneration syndromes. J. Neurol. 267, 2228–2238. doi: 10.1007/s00415-020-09824-1, PMID: 32277260 PMC7359154

[ref122] Nagahama Y. Okina T. Suzuki N. Matsuda M. (2006). The Cambridge behavioral inventory: validation and application in a memory clinic. J. Geriatr. Psychiatry Neurol. 19, 220–225. doi: 10.1177/0891988706286545, PMID: 17085761

[ref123] Neary D. Snowden J. S. Gustafson L. Passant U. Stuss D. Black S. . (1998). Frontotemporal lobar degeneration: a consensus on clinical diagnostic criteria. Neurology 51, 1546–1554. doi: 10.1212/WNL.51.6.1546 9855500

[ref124] O’Connor C. M. Clemson L. Hornberger M. Leyton C. E. Hodges J. R. Piguet O. . (2016). Longitudinal change in everyday function and behavioral symptoms in frontotemporal dementia. Neurol. Clin. Pract. 6, 419–428. doi: 10.1212/CPJ.0000000000000264, PMID: 27847684 PMC5100706

[ref125] O’Connor C. M. Landin-Romero R. Clemson L. Kaizik C. Daveson N. Hodges J. R. . (2017). Behavioral-variant frontotemporal dementia: distinct phenotypes with unique functional profiles. Neurology 89, 570–577. doi: 10.1212/WNL.0000000000004215, PMID: 28701492 PMC5562953

[ref126] Oeckl P. Anderl-Straub S. Von Arnim C. A. F. Baldeiras I. Diehl-Schmid J. Grimmer T. . (2022). Serum GFAP differentiates Alzheimer’s disease from frontotemporal dementia and predicts MCI-to-dementia conversion. J. Neurol. Neurosurg. Psychiatry 93, 659–667. doi: 10.1136/jnnp-2021-328547, PMID: 35477892

[ref127] Otten R. De Vries R. Schoonmade L.. (2019). Amsterdam efficient deduplication (AED) method (version 1). Available at: https://zenodo.org/records/3582928

[ref128] Ouzzani M. Hammady H. Fedorowicz Z. Elmagarmid A. (2016). Rayyan—a web and mobile app for systematic reviews. Syst. Rev. 5:210. doi: 10.1186/s13643-016-0384-4 27919275 PMC5139140

[ref129] Page M. J. Mckenzie J. E. Bossuyt P. M. Boutron I. Hoffmann T. C. Mulrow C. D. . (2021). The PRISMA 2020 statement: an updated guideline for reporting systematic reviews. BMJ 372:N71. doi: 10.1136/bmj.n71 33782057 PMC8005924

[ref130] Pérez-Millan A. Contador J. Juncà-Parella J. Bosch B. Borrell L. Tort-Merino A. . (2023). Classifying Alzheimer’s disease and frontotemporal dementia using machine learning with cross-sectional and longitudinal magnetic resonance imaging data. Hum. Brain Mapp. 44, 2234–2244. doi: 10.1002/hbm.26205, PMID: 36661219 PMC10028671

[ref131] Pfeffer R. I. Kurosaki T. T. Harrah C. H. Jr. Chance J. M. Filos S. (1982). Measurement of functional activities in older adults in the community. J. Gerontol. 37, 323–329. doi: 10.1093/geronj/37.3.323 7069156

[ref132] Poos J. M. Jiskoot L. C. Leijdesdorff S. M. J. Seelaar H. Panman J. L. Van Der Ende E. L. . (2020). Cognitive profiles discriminate between genetic variants of behavioral frontotemporal dementia. J. Neurol. 267, 1603–1612. doi: 10.1007/s00415-020-09738-y, PMID: 32052166 PMC7293665

[ref133] Premi E. Gualeni V. Costa P. Cosseddu M. Gasparotti R. Padovani A. . (2016). Looking for measures of disease severity in the frontotemporal dementia continuum. J. Alzheimers Dis. 52, 1227–1235. doi: 10.3233/JAD-160178, PMID: 27104906

[ref134] Radakovic R. Abrahams S. (2014). Developing a new apathy measurement scale: dimensional apathy scale. Psychiatry Res. 219, 658–663. doi: 10.1016/j.psychres.2014.06.010, PMID: 24972546

[ref135] Ramanan S. Bertoux M. Flanagan E. Irish M. Piguet O. Hodges J. R. . (2017). Longitudinal executive function and episodic memory profiles in behavioral-variant frontotemporal dementia and Alzheimer’s disease. J. Int. Neuropsychol. Soc. 23, 34–43. doi: 10.1017/S1355617716000837, PMID: 27751195

[ref136] Ranasinghe K. G. Rankin K. P. Lobach I. V. Kramer J. H. Sturm V. E. Bettcher B. M. . (2016). Cognition and neuropsychiatry in behavioral variant frontotemporal dementia by disease stage. Neurology 86, 600–610. doi: 10.1212/WNL.0000000000002373, PMID: 26802093 PMC4762418

[ref137] Ranasinghe K. G. Toller G. Cobigo Y. Chiang K. Callahan P. Eliazer C. . (2021). Computationally derived anatomic subtypes of behavioral variant frontotemporal dementia show temporal stability and divergent patterns of longitudinal atrophy. Alzheimers Dement. 13:e12183. doi: 10.1002/dad2.12183, PMID: 34268446 PMC8274310

[ref138] Rascovsky K. Hodges J. R. Knopman D. Mendez M. F. Kramer J. H. Neuhaus J. . (2011). Sensitivity of revised diagnostic criteria for the Behavioural variant of frontotemporal dementia. Brain 134, 2456–2477. doi: 10.1093/brain/awr179, PMID: 21810890 PMC3170532

[ref139] Reus L. M. Vijverberg E. G. Tijms B. M. Kate M. T. Gossink F. Krudop W. A. . (2018). Disease trajectories in behavioural variant frontotemporal dementia, primary psychiatric and other neurodegenerative disorders presenting with behavioural change. J. Psychiatr. Res. 104, 183–191. doi: 10.1016/j.jpsychires.2018.07.014, PMID: 30103065

[ref140] Roveta F. Marcinnò A. Cremascoli R. Priano L. Cattaldo S. Rubino E. . (2022). Increased orexin a concentrations in cerebrospinal fluid of patients with behavioural variant frontotemporal dementia. Neurol. Sci. 43, 313–317. doi: 10.1007/s10072-021-05250-x, PMID: 33904007 PMC8724071

[ref141] Santacruz Escudero J. M. Beltran J. Palacios A. Chimbi C. M. Matallana D. Reyes P. . (2019). Neuropsychiatric symptoms as predictors of clinical course in neurodegeneration. A longitudinal study. Front. Aging Neurosci. 11:176. doi: 10.3389/fnagi.2019.00176, PMID: 31396074 PMC6668630

[ref142] Scherling C. S. Hall T. Berisha F. Klepac K. Karydas A. Coppola G. . (2014). Cerebrospinal fluid neurofilament concentration reflects disease severity in frontotemporal degeneration. Ann. Neurol. 75, 116–126. doi: 10.1002/ana.24052, PMID: 24242746 PMC4020786

[ref143] Schubert S. Leyton C. E. Hodges J. R. Piguet O. (2016). Longitudinal memory profiles in behavioral-variant frontotemporal dementia and Alzheimer’s disease. J. Alzheimers Dis. 51, 775–782. doi: 10.3233/JAD-150802 26890749

[ref144] Seeley W. W. Crawford R. Rascovsky K. Kramer J. H. Weiner M. Miller B. L. . (2008). Frontal paralimbic network atrophy in very mild behavioral variant frontotemporal dementia. Arch. Neurol. 65, 249–255. doi: 10.1001/archneurol.2007.38, PMID: 18268196 PMC2544627

[ref145] Shaw A. D. Hughes L. E. Moran R. Coyle-Gilchrist I. Rittman T. Rowe J. B. (2019). *In vivo* assay of cortical microcircuitry in frontotemporal dementia: a platform for experimental medicine studies. Cereb. Cortex 31:1837. doi: 10.1093/cercor/bhz024 PMC7869085 31216360

[ref146] Sheelakumari R. Venkateswaran R. Chandran A. Varghese T. Zhang L. Yue G. H. . (2018). Quantitative analysis of grey matter degeneration in FTD patients using fractal dimension analysis. Brain Imaging Behav. 12, 1221–1228. doi: 10.1007/s11682-017-9784-x 29086152

[ref147] Shigenobu K. Ikeda M. Fukuhara R. Maki N. Hokoishi K. Nebu A. . (2002). The stereotypy rating inventory for frontotemporal lobar degeneration. Psychiatry Res. 110, 175–187. doi: 10.1016/S0165-1781(02)00094-X, PMID: 12057829

[ref148] Singleton E. Fieldhouse J. Hooft J. Scarioni M. Engelen M.-P. Sikkes S. . (2022). Social cognition deficits and biometric signatures in the behavioural variant of Alzheimer’s disease. Brain 146, 1163–2174. doi: 10.1093/brain/awac382 PMC10151185 36268579

[ref149] Smits L. L. Van Harten A. C. Pijnenburg Y. A. Koedam E. L. Bouwman F. H. Sistermans N. . (2015). Trajectories of cognitive decline in different types of dementia. Psychol. Med. 45, 1051–1059. doi: 10.1017/S0033291714002153 25229325

[ref150] Spotorno N. Lindberg O. Nilsson C. Landqvist Waldö M. Van Westen D. Nilsson K. . (2020). Plasma neurofilament light protein correlates with diffusion tensor imaging metrics in frontotemporal dementia. PLoS One 15:e0236384. doi: 10.1371/journal.pone.0236384, PMID: 33108404 PMC7591030

[ref151] Staffaroni A. M. Bajorek L. Casaletto K. B. Cobigo Y. Goh S. M. Wolf A. . (2019a). Assessment of executive function declines in presymptomatic and mildly symptomatic familial frontotemporal dementia: NIH-Examiner as a potential clinical trial endpoint. Alzheimers Dement. 16, 11–21. doi: 10.1016/j.jalz.2019.01.012 PMC6842665 31914230

[ref152] Staffaroni A. M. Ljubenkov P. A. Kornak J. Cobigo Y. Datta S. Marx G. . (2019b). Longitudinal multimodal imaging and clinical endpoints for frontotemporal dementia clinical trials. Brain 142, 443–459. doi: 10.1093/brain/awy319, PMID: 30698757 PMC6351779

[ref153] Staffaroni A. M. Quintana M. Wendelberger B. Heuer H. W. Russell L. L. Cobigo Y. . (2022). Temporal order of clinical and biomarker changes in familial frontotemporal dementia. Nat. Med. 28, 2194–2206. doi: 10.1038/s41591-022-01942-9, PMID: 36138153 PMC9951811

[ref154] Steinacker P. Anderl-Straub S. Diehl-Schmid J. Semler E. Uttner I. Von Arnim C. A. F. . (2018). Serum neurofilament light chain in behavioral variant frontotemporal dementia. Neurology 91, e1390–e1401. doi: 10.1212/WNL.0000000000006318, PMID: 30209235

[ref155] Tan K. S. Libon D. J. Rascovsky K. Grossman M. Xie S. X. (2013). Differential longitudinal decline on the mini-mental state examination in frontotemporal lobar degeneration and Alzheimer disease. Alzheimer Dis. Assoc. Disord. 27, 310–315. doi: 10.1097/WAD.0b013e31827bdc6f, PMID: 23314064 PMC3648632

[ref156] Tavares T. P. Mitchell D. G. V. Coleman K. K. Coleman B. L. Shoesmith C. L. Butler C. R. . (2020). Early symptoms in symptomatic and preclinical genetic frontotemporal lobar degeneration. J. Neurol. Neurosurg. Psychiatry 91, 975–984. doi: 10.1136/jnnp-2020-322987, PMID: 32769115 PMC7611534

[ref157] Toller G. Mandelli M. L. Cobigo Y. Rosen H. J. Kramer J. H. Miller B. L. . (2022). Right uncinate fasciculus supports socioemotional sensitivity in health and neurodegenerative disease. NeuroImage Clin. 34:102994. doi: 10.1016/j.nicl.2022.102994, PMID: 35487131 PMC9125782

[ref158] Toller G. Ranasinghe K. Cobigo Y. Staffaroni A. Appleby B. Brushaber D. . (2020). Revised self-monitoring scale: a potential endpoint for frontotemporal dementia clinical trials. Neurology 94, e2384–e2395. doi: 10.1212/WNL.0000000000009451, PMID: 32371446 PMC7357291

[ref159] Torralva T. Gleichgerrcht E. Torres Ardila M. J. Roca M. Manes F. F. (2015). Differential cognitive and affective theory of mind abilities at mild and moderate stages of behavioral variant frontotemporal dementia. Cogn. Behav. Neurol. 28, 63–70. doi: 10.1097/WNN.0000000000000053, PMID: 26102996

[ref160] Tsai R. M. Bejanin A. Lesman-Segev O. LaJoie R. Visani A. Bourakova V. . (2019). ^18^F-flortaucipir (Av-1451) Tau pet in frontotemporal dementia syndromes. Alzheimers Res. Ther. 11:13. doi: 10.1186/s13195-019-0470-7, PMID: 30704514 PMC6357510

[ref161] Vieira D. Durães J. Baldeiras I. Santiago B. Duro D. Lima M. . (2019). Lower CSF amyloid-beta_1–42_ predicts a higher mortality rate in frontotemporal dementia. Diagnostics 9:162. doi: 10.3390/diagnostics9040162 31731494 PMC6963225

[ref162] Vijverberg E. G. Dols A. Krudop W. A. Peters A. Kerssens C. J. Van Berckel B. N. . (2016). Diagnostic accuracy of the frontotemporal dementia consensus criteria in the late-onset frontal lobe syndrome. Dement. Geriatr. Cogn. Disord. 41, 210–219. doi: 10.1159/000444849, PMID: 27160162

[ref163] Walia N. Eratne D. Loi S. M. Li Q. X. Varghese S. Malpas C. B. . (2022). Cerebrospinal fluid Neurofilament light predicts the rate of executive function decline in younger-onset dementia. J. Neurol. Sci. 432:120088. doi: 10.1016/j.jns.2021.120088, PMID: 34922179

[ref164] Wear H. J. Wedderburn C. J. Mioshi E. Williams-Gray C. H. Mason S. L. Barker R. A. . (2008). The Cambridge behavioural inventory revised. Dement. Neuropsychol. 2, 102–107. doi: 10.1590/S1980-57642009DN20200005, PMID: 29213551 PMC5619578

[ref165] Wei G. Irish M. Hodges J. R. Piguet O. Kumfor F. (2020). Disease-specific profiles of apathy in Alzheimer’s disease and behavioural-variant frontotemporal dementia differ across the disease course. J. Neurol. 267, 1086–1096. doi: 10.1007/s00415-019-09679-1, PMID: 31873787

[ref166] Whitwell J. L. Tosakulwong N. Schwarz C. C. Senjem M. L. Spychalla A. J. Duffy J. R. . (2020). Longitudinal anatomic, functional, and molecular characterization of pick disease phenotypes. Neurology 95, e3190–e3202. doi: 10.1212/WNL.0000000000010948, PMID: 32989107 PMC7836669

[ref167] Wicklund A. H. Rademaker A. Johnson N. Weitner B. B. Weintraub S. (2007). Rate of cognitive change measured by neuropsychologic test performance in 3 distinct dementia syndromes. Alzheimer Dis. Assoc. Disord. 21, S70–S78. doi: 10.1097/WAD.0b013e31815bf8a5, PMID: 18090428

[ref168] Woollacott I. O. C. Nicholas J. M. Heslegrave A. Heller C. Foiani M. S. Dick K. M. . (2018). Cerebrospinal fluid soluble TREM2 levels in frontotemporal dementia differ by genetic and pathological subgroup. Alzheimers Res. Ther. 10:79. doi: 10.1186/s13195-018-0405-8, PMID: 30111356 PMC6094471

[ref169] Woolley J. D. Khan B. K. Murthy N. K. Miller B. L. Rankin K. P. (2011). The diagnostic challenge of psychiatric symptoms in neurodegenerative disease: rates of and risk factors for prior psychiatric diagnosis in patients with early neurodegenerative disease. J. Clin. Psychiatry 72, 126–133. doi: 10.4088/JCP.10m06382oli, PMID: 21382304 PMC3076589

[ref170] Yu J. Lee T. M. C. (2019). The longitudinal decline of white matter microstructural integrity in behavioral variant frontotemporal dementia and its association with executive function. Neurobiol. Aging 76, 62–70. doi: 10.1016/j.neurobiolaging.2018.12.005, PMID: 30703627

