# Supplementary material for: The pursuit for markers of disease progression in behavioral variant frontotemporal dementia: a scoping review to optimize outcome measures for clinical trials
Source: Front Aging Neurosci. 2024 May 9;16:1382593. doi: 10.3389/fnagi.2024.1382593 (PMC11112081; doi:10.3389/fnagi.2024.1382593)
Supplement: Supplementary file 1 [file Table_1.docx]

### **Supplementary information S1**

###

### PubMed Search History September 5, 2022

| **Search** | **PubMed Query – September 5, 2022** | **Items found** |
| --- | --- | --- |
| #4 | #1 AND #2 AND #3 | 2,245 |
| #3 | "Cohort Studies"[Mesh] OR "Disease Progression"[Mesh] OR longitudinal[tiab] OR follow-up[tiab] OR prospective[tiab] OR retrospective[tiab] OR stage*[tiab] OR course[tiab] OR severit*[tiab] OR progressi*[tiab] OR change*[tiab] OR deteriorate*[tiab] OR exacerbation*[tiab] OR decline[tiab] OR traject*[tiab] OR rate[tiab] | 9,828,777 |
| #2 | behavioural[tiab] OR behavioral[tiab] OR "frontal variant*"[tiab] | 439,114 |
| #1 | bvFTD*[tiab] OR fvFTD*[tiab] OR ("Frontotemporal Lobar Degeneration"[Mesh:NoExp] OR "Frontotemporal Dementia"[Mesh] OR "frontotemporal lobar degenerat*" [tiab] OR "frontotemporal degenerat*"[tiab] OR "frontotemporal dementia*"[tiab] OR "frontotemporal lobe dementia*"[tiab] OR "pick’s disease*"[tiab] OR FTLD*[tiab] OR FTD[tiab] OR FTDs[tiab]) | 13,293 |

### Embase.com Search History September 5, 2022

| **Search** | **Embase.com Query – September 5, 2022** | **Items found** |
| --- | --- | --- |
| #6 | #4 NOT ('conference abstract'/it OR 'conference review'/it) | 2,686 |
| #5 | #4 AND ('conference abstract'/it OR 'conference review'/it) | 1,859 |
| #4 | #1 AND #2 AND #3 | 4,555 |
| #3 | 'cohort analysis'/exp OR 'disease course'/exp OR 'longitudinal study'/exp OR 'prospective study'/exp OR 'retrospective study'/exp OR 'follow up'/exp OR 'disease severity'/exp OR longitudinal:ab,ti,kw OR follow-up:ab,ti,kw OR prospective:ab,ti,kw OR retrospective:ab,ti,kw OR stage*:ab,ti,kw OR course:ab,ti,kw OR severit*:ab,ti,kw OR progressi*:ab,ti,kw OR change*:ab,ti,kw OR deteriorate*:ab,ti,kw OR exacerbation*:ab,ti,kw OR decline:ab,ti,kw OR traject*:ab,ti,kw OR rate*:ab,ti,kw | 15,476,411 |
| #2 | behavioural:ab,ti,kw OR behavioral:ab,ti,kw OR (frontal NEAR/3 variant*):ab,ti,kw | 530,743 |
| #1 | bvFTD*:ab,ti,kw OR fvFTD*:ab,ti,kw OR ('frontotemporal dementia'/de OR 'frontal variant frontotemporal dementia'/exp OR (frontotemporal NEAR/3 degenerat*):ab,ti,kw OR (frontotemporal NEAR/3 dementia*):ab,ti,kw OR ‘pick* disease*’:ab,ti,kw OR FTLD*:ab,ti,kw OR FTD*:ab,ti,kw) | 27,005 |
